# CaLB Catalyzed Conversion of ε-Caprolactone in Aqueous Medium. Part 1: Immobilization of CaLB to Microgels

**DOI:** 10.3390/polym8100372

**Published:** 2016-10-19

**Authors:** Stefan Engel, Heidi Höck, Marco Bocola, Helmut Keul, Ulrich Schwaneberg, Martin Möller

**Affiliations:** 1 DWI—Leibniz Institute for Interactive Materials and Institute of Technical and Macromolecular Chemistry, RWTH Aachen University, Forckenbeckstraße 50, D-52056 Aachen, Germany; Engel@dwi.rwth-aachen.de (S.E.); Keul@dwi.rwth-aachen.de (H.K.); 2 DWI—Leibniz Institute for Interactive Materials and Institute of Biotechnology, RWTH Aachen University, Forckenbeckstraße 50, D-52056 Aachen, Germany; Hoeck@dwi.rwth-aachen.de (H.H.); M.Bocola@biotec.rwth-aachen.de (M.B.); U.Schwaneberg@biotec.rwth-aachen.de (U.S.)

**Keywords:** enzyme immobilization, microgel, *Candida antarctica* lipase B, ε-Caprolactone, Novozym^®^ 435, polyesterification

## Abstract

The enzymatic ring-opening polymerization of lactones is a method of increasing interest for the synthesis of biodegradable and biocompatible polymers. In the past it was shown that immobilization of *Candida antarctica* lipase B (CaLB) and the reaction medium play an important role in the polymerization ability especially of medium ring size lactones like ε-caprolactone (ε-CL). We investigated a route for the preparation of compartmentalized microgels based on poly(glycidol) in which CaLB was immobilized to increase its esterification ability. To find the ideal environment for CaLB, we investigated the acceptable water concentration and the accessibility for the monomer in model polymerizations in toluene and analyzed the obtained oligomers/polymers by NMR and SEC. We observed a sufficient accessibility for ε-CL to a toluene like hydrophobic phase imitating a hydrophobic microgel. Comparing free CaLB and Novozym^®^ 435 we found that not the monomer concentration but rather the solubility of the enzyme, as well as the water concentration, strongly influences the equilibrium of esterification and hydrolysis. On the basis of these investigations, microgels of different polarity were prepared and successfully loaded with CaLB by physical entrapment. By comparison of immobilized and free CaLB, we demonstrated an effect of the hydrophobicity of the microenvironment of CaLB on its enzymatic activity.

## 1. Introduction

The demand for biocompatible and biodegradable materials like polyesters has been greatly increased during the last decades especially due to its versatile applications in the fields of (bio)medicine and tissue engineering [1,2]. For these applications it is a special challenge of our time to substitute organic solvents as reaction medium with “green” solvents like water [3]. Enzymes in general and lipases in particular are specialized for reactions in aqueous medium. In natural environment lipases are known to hydrolyze fats at the lipid-water interface with formation of fatty acids and glycerol [4,5]. In organic solvents it was shown that lipases also catalyze esterification reactions [6] with the formation of polyesters [7]. In order to reduce the use of ecologically harmful organic solvents, which are always found in traces in the final products, we attempt to engineer the micro surrounding of the lipase such, that in the future it can be used for the catalysis of polyesterifications in pure water. In the literature, only a few reports can be found on this topic [8,9,10,11].

In the field of organic chemistry, the enzymatic (trans) esterification is widely studied since the 1980s [12,13], whereas in polymer chemistry lipases were under investigation for the first time in 1993. The groups of Kobayashi and Kohn reconnoitered independently the mechanism of the enzyme catalyzed ring-opening polymerization (ROP) of cyclic esters [14,15]. In the following years, Kobayashi et al. further compared the esterification ability of macrolides and smaller ring size lactones like ε-CL with various lipases in organic solvent. It was found that macrolides show a much higher reactivity and degree of polymerization compared to ε-CL [16]. This behavior shows an opposing trend in metal catalyzed ROP, where high molecular weights were obtained for lactones with medium ring size while macrolides were less reactive [17,18,19].

The first few experiments to perform polyesterifications in water were also reported in the 1990s. It was observed that macrolides in contrast to ε-CL can be polymerized due to their insolubility in water. The formation of a monomer emulsion leads to compartmentalization of the reaction medium comparable to nature, where the compartmentalization of a cell enables the enzymatic production of polynucleotides and peptides [10,11]. In the miniemulsion the water concentration at the interface (where the lipase is expected to attach, due to its hydrophobic surface around the active site), as well as in the monomer phase, is highly limited and the esterification is the preferred catalyzed reaction of the lipase. Though, compared to the reaction in pure organic solvents the obtained degree of polymerization for macrolides is much lower, e.g., the polymerization of 11-undecanolide (UDL) in water yielded the corresponding polyester with 79% conversion and *M*_n_ 1300 Da [8]. However, in the case of water miscible lactones like ε-CL, no emulsions are formed and no compartmentalization occurs. Due to the high water concentration in the close environment of both the active site of the lipase and the monomer, hydrolysis of the lactone ring yielding 6-hydroxycaproic acid is the preferably catalyzed reaction. These results show that by changing the microenvironment of the enzyme and the substrate, the catalyzed synthesis can be directed to the desired product. In this case, the hydrolysis-esterification equilibrium can be pushed towards the esterification by using more hydrophobic monomers. In synthetic chemistry the use of miniemulsions as nanoreactors for directing enzymatic catalyzed reactions is a common method, not only for the synthesis of linear aliphatic polyesters [20], but also for the aminolysis of lactones [21] or the preparation of biodegradable pentadecalactone (PDL) nanoparticles [22] among others [23].

When talking about directing of enzymatic catalysis, immobilization is another important method, bringing various benefits like efficient recovery, improving process economy as well as stabilization and protection of the enzyme against exterior influences like pH, solvents or temperature. Especially protection of the enzyme is a very important point regarding the effect of water on the esterification ability of lipases described above. A widely used and commercially available immobilized lipase is *Candida antarctica* lipase B (CaLB) adsorbed on a macroporous acrylic resin (Novozym^®^ 435). Next to its application for aminolysis or transesterification it is the most often used lipase formulation for the enzymatic ROP of lactones in organic solvents and was well studied by various groups [24,25]. Using Novozym^®^ 435 the obtained number average molecular weight *M*_n_ of poly(ε-caprolactone) (P(ε-CL)) is between 10 and 25 kDa if the polymerization is performed in dry organic media. To the best of our knowledge, this high polymerization ability of CaLB has never been reached by any other immobilized CaLB catalyst and therefore Novozym^®^ 435 can be seen as a benchmark for polyesterification in organic solvents. On the other hand, using Novozym^®^ 435 in water again the hydrolysis of the ester is the preferred catalyzed reaction and only low molecular weight oligomers are obtained. The reason for the great performance of Novozym^®^ 435 in organic solvents and the low degree of polymerization in water has never been discussed in literature so far and will be taken up in the publication in hand. At this point it should be mentioned that by working with Novozym^®^ 435 in water we detected traces of a nonionic poly(ethylene oxide/propylene oxide) copolymer surfactant in the reaction products (unpublished results). To our knowledge the presence of the surfactant in Novozym^®^ 435 and its role on the activity of CaLB has never been explained, however, this observation will not be subject of the present manuscript.

In this publication we will thus focus on the effect of different solvent compositions and of the immobilization of CaLB in microgels by physical entrapment on the hydrolytic and esterification activity. The poly(glycidol) based microgels we will use were developed by our group recently [26,27]. They provide, in terms of hydrophilic-lipophilic balance, an adjustable and compartmentalized structure and therefore offer a high concentration of interfaces, where the polymerization is expected to take place. The reaction products—oligomers and polymers—are analyzed by NMR and SEC measurements. By analyzing the ratio of hydrolysis and esterification product we will investigate the esterification ability of CaLB under varying reaction conditions. Further we will analyze the dispersity of the polymeric products and discuss the activation of the lipase by immobilization as well as the possible coexistence of different active “forms” of CaLB (aggregated, soluble, bound, protected, inhibited) according to the classification described by Fernández-Lafuente et al. [28].

## 2. Materials and Methods

### 2.1. Materials and Instrumentation

ε-Caprolactone (99%, Alfa Aesar, Karlsruhe, Germany), allyl glycidyl ether (AGE, >99%, Sigma Aldrich Chemie GmbH, Steinheim, Germany), *tert*-butyl glycidyl ether (*t*BGE, 99%, Sigma Aldrich Chemie GmbH, Steinheim, Germany), toluene (VWR Chemicals, Fontenay-sous-Bais, France), caesium hydroxide (96%, Alfa Aesar, Karlsruhe, Germany), 2,2-dimethoxy-2-phenylacetophenone (DMPA, 99%, Sigma Aldrich Chemie GmbH, Steinheim, Germany), sodium dodecyl sulfate (SDS, Bio-Rad Laboratories GmbH, München, Germany), hexadecane (99%, Sigma Aldrich Chemie GmbH, Steinheim, Germany), 2,2′-(ethylenedioxy)diethanethiol (95%, Sigma Aldrich, Quimica, Toluca, Mexico), triethylene glycol monomethyl ether (TEGME, >97%, Merck, Darmstadt, Germany), calcium hydride (93%, Thermofisher Acros Organics, Geel, Belgium), ethyl vinyl ether (99%, Sigma Aldrich Chemie GmbH, Steinheim,, Germany) and glycidol (96%, Sigma Aldrich Chemie GmbH, Steinheim, Germany) were used as received. Ethoxyethyl glycidyl ether (EEGE) was prepared according to Fitton et al. [29]. Toluene, *tert*-butyl glycidyl ether, allyl glycidyl ether, ethoxyethyl glycidyl ether and ε-caprolactone were dried over CaH_2_ for 24 h and distilled under nitrogen atmosphere before use. Novozym^®^ 435 (Novozymes A/S, Bagsvaerd, Denmark) was used as received, while CaLB-wt was obtained from c-LEcta (Leipzig, Germany) and purified or produced by ourselves. The protocol for the cloning [30], production and purification of CaLB is attached in the supplementary materials (Section 8).

^1^H- and ^13^C-NMR spectra were recorded on a Bruker Avance III 400 spectrometer (400 MHz and 100 MHz, respectively; Bruker Corporation, Billerica, MA, USA) and are reported as follows: chemical shift δ (ppm) (multiplicity, number of protons, assignment). Chloroform (CDCl_3_, δH = 7.26 ppm, δC = 77.0 ppm) was used as an internal standard. Chemical shifts are reported in ppm to the nearest 0.01 ppm for ^1^H- and the nearest 0.1 ppm for ^13^C-NMR. The ^1^H-NMR spectra for the determination of the partition coefficient of ε-CL were recorded on a Bruker Avance I 600 spectrometer (600 MHz). Water (D_2_O, δH = 4.79 ppm) and toluene (toluene-d_8_, δH = 2.09 ppm) were used as internal standard. Molecular weights (*M*_n_ and *M*_w_) and molecular weight distributions (*Ð*) were determined by size-exclusion chromatography (SEC). The results were evaluated using the PSS WinGPC UniChrom software (Version 8.1.1). SEC analyses were carried out with tetrahydrofuran (THF, HPLC grade, Carl Roth, Karlsruhe, Germany) as eluent using a HPLC pump (PU-2080plus, Jasco, Gross-Umstadt, Germany) equipped with a refractive index detector (RI-2031plus, Jasco, Gross-Umstadt, Germany). The sample solvent contained 250 mg∙mL^−1^ 3,5-*di*-*tert*-4-butylhydroxytoluene (BHT, ≥99%, Sigma Aldrich Chemie GmbH, Steinheim, Germany) as internal standard. One pre-column (8 mm × 50 mm) and four SDplus gel columns (8 mm × 300 mm, SDplus, MZ Analysentechnik, Mainz, Germany) were applied at a flow rate of 1.0 mL∙min^−1^ at 20 °C. The diameter of the gel particles measured 5 µm, the nominal pore widths were 50, 10^2^, 10^3^ and 10^4^ Å. Calibration was achieved using narrow distributed poly(methyl methacrylate) standards (PSS Polymer Standards Service GmbH, Mainz, Germany) from 600 to 576,000 Da.

The hydrolytic activity of the free and immobilized CaLB was determined by triple determination by a *para*-nitrophenyl butyrate *(pNPB)* assay. The microgel sample was diluted to linear range of the assay. The activity was determined by addition of triethanolamine (TEA) buffer (90 µL, 0.1 M, pH 7.5) to the diluted sample (10 µL) and freshly prepared substrate solution (TEA buffer (100 µL) containing *p*NPB (0.5 mM) and acetonitrile (10%, *v*/*v*)). The release of *para*-nitrophenolate was recorded by measuring the absorption at 410 nm at room temperature over 8 min on a microtiter plate reader (Omega FLUOstar, BMG LABTECH, Ortenberg, Germany).

The amino acid analysis was performed after hydrolysis of the enzyme by capillary-gas liquid chromatography with mass-spectrometry coupling (GC-MS) (triple determination). Therefore the CaLB loaded microgel (1 mL) was mixed with conc. HCl (1:1 *v*/*v*) in an ampoule. After addition of a 1 M phenole solution in 6 M HCl (20 µL) the mixture was degassed, the ampoule sealed under vacuum and heated to 110 °C for 24 h. The aqueous solution was decanted from the microgel residues and the water was removed by evaporation. The hydrolysates were washed twice with water, before they were incubated in pyridine (1 mL) at 60 °C. The cooled solutions were centrifuged (1 min, 13,000 rpm) twice and the respective supernatants were transferred in a volumetric flask. The combined collected supernatants were diluted with pyridine to a final volume of 2 mL. For the preparation of the *tert*-butyldimethylsilyl (TBDMS) derivates the hydrolysate solution (200 µL) was mixed with *N*-methyl-*N*-*tert*-butyldimethylsilyl-trifluoroacetamide (MBDSTFA, 400 µL) and pyridine (400 µL) and heated for 1 h at 60 °C. After cooling to room temperature the samples were analyzed by GC-MS.

GC-MS was performed at a Perkin Elmer Clarus 600 Mass Spectrometer (Perkin Elmer Inc., Waltham, MA, USA). Aliquots of 1 µL of the amino acid TBDMS solutions were injected at 280 °C and chromatographed on a Phenomenex 5% diphenyl/95% dimethyl polysiloxane MS capillary (30 m, 250 µm ID, 0.25 µm film). The initial oven temperature was 80 °C and a temperature gradient with a heating rate of 3 °C/min up to 300 °C was run. The carrier gas, helium, was set to 1 mL/min with 10 mL/min split at the injector port. The mass device was operated in the full scan mode with electron impact ionization and masses recorded from 45 to 620 Da; the transfer line was held at 200 °C and the source temperature at 240 °C.

### 2.2. Partition Coefficient of ε-CL in D_2_O/Toluene-d_8_

D_2_O (1 mL), toluene-d_8_ (1 mL) and ε-CL (80 µL) were mixed in a 5 mL glass vial with screw cap. The exact masses of all components were weighed and are shown in Table S1. The mixtures were heated for 12 h at various temperatures (*T* = 25, 35, 45 and 55 °C) under vigorous stirring. Samples of 500 µL were taken out of both phases at the particular temperature and weighed in a NMR tube. As a standard, dioxane (8 µL) was added to each NMR tube. The ^1^H-NMR spectra were measured at the respective temperatures. ^1^H-NMR (600 MHz, D_2_O) (Figure S1): δ = 4.38–4.36 (m, 2H, –C*H*_2_O–), 3.77 (s, 8H, dioxane), 2.72–2.70 (m, 2H, –C*H*_2_CO–), 1.87–1.84 (m, 2H, –C*H*_2_CH_2_O–), 1.81–1.77 (m, 2H, –C*H*_2_CH_2_CO–), 1.75–1.72 (m, 2H, –CH_2_C*H*_2_CH_2_–) ppm. ^1^H-NMR (600 MHz, toluene-*d*_8_) (Figure S2): δ = 3.58–3.57 (m, 2H, –C*H*_2_O–), 3.35 (s, 8H, dioxane), 2.17–2.15 (m, 2H, –C*H*_2_CO–), 1.27–1.23 (m, 2H, –C*H*_2_CH_2_O–), 1.23–1.20 (m, 2H, –C*H*_2_CH_2_CO–), 1.15–1.11 (m, 2H, –CH_2_C*H*_2_CH_2_–) ppm.

### 2.3. Enzymatic Polymerization of ε-CL in Water, Toluene and Bulk

In a 25 mL flame dried Schlenk tube free CaLB (1 mg) was dissolved in a mixture of ε-CL (water content: 1532 ppm) and toluene (water content: 266 ppm) and H_2_O respectively. The overall mass was 4 g in which the mass ratio of the components was varied as shown in Table S2. After stirring the solutions for 46 h at 50 °C the solvent was removed and the product dried in vacuum. The product was analyzed without further purification. ^1^H-NMR (400 MHz, D_2_O) (Figure S4): δ = 4.16–4.14 (t, 2H, 1a), 3.99–3.95 (t, 2H, 1c), 3.56–3.53 (t, 4H, 1b), 2.57–2.54 (t, 2H, 2a), 2.27–2.20 (m, 4H, 2b, 2c), 1.77 (m, 2H, 3a), 1.68 (m, 4H, 4a + 5a), 1.56 (m, 8H, 3b, 3c, 4b, 4c), 1.30 (m, 4H, 5b, 5c) ppm. Exemplarily the SEC traces of sample No. 2.4 and 2.7 of Table S2 are shown in Figure S3.

### 2.4. Enzymatic Polymerization of ε-CL in Toluene with Varying Water Concentrations

In a 25 mL flame dried Schlenk tube free CaLB (1 mg) or Novozym^®^ 435 (10 mg, 1/10 wt/wt) was dispersed in a mixture of anhydrous and water saturated toluene in different ratios with an overall volume of 3.2 g (see Tables S3–S8). ε-CL monomer (0.8 g) was added and the solutions were stirred for 46 h at 50 °C. The solvent was evaporated and the product was dried in vacuum. The product was analyzed by NMR and SEC without further purification.

### 2.5. Synthesis of P(EEGE)_0.8_-b-P(AGE)_0.2_ 1

Caesium hydroxide (0.175 g, 1.03 mmol, 1/25 eq.) and triethylene glycol monomethylether (0.170 g, 1.03 mmol, 1/25 eq.) were dissolved in benzene (5 mL) and stirred at 60 °C for 30 min. The benzene/water azeotrope was removed by distillation at 90 °C and the residue was dried for another 3 h at 90 °C. Ethoxyethyl glycidyl ether (2.90 g, 19.83 mmol, 0.8 eq.) was added at room temperature and the reaction mixture was heated to 40 °C. After 17 h allyl glycidyl ether (0.554 g, 4.85 mmol, 0.2 eq.) was added and the temperature was increased to 50 °C. After 24 h and full conversion the polymerization was terminated by the addition of ethanol (1 mL). The polymer solution in ethanol was precipitated in cold water, filtrated and dried in vacuum. The product was obtained as orange oil. Yield 2.34 g (68%). ^1^H-NMR (400 MHz, CDCl_3_) (Figure S5): δ = 5.86 (m, 1H, –OCH_2_C*H*CH_2_), 5.26–5.13 (dd, 2H, –OCH_2_CHC*H*_2_), 4.68 (m, 1H, CH_3_C*H*–), 3.96 (d, 2H, –OC*H*_2_CHCH_2_), 3.63–3.44 (m, 12H, –C*H*_2_CHO–_(backbone)_, –CH_2_C*H*O–_(backbone)_, –OCH_2_CH_(backbone)_C*H*_2_O–, –OC*H*_2_CH_3_), 3.35 (s, 3H, –O–C*H*_3__(Initiator)_) 1.28–1.27 (d, 3H, –CHC*H*_3_), 1.18 (t, 3H, –OCH_2_C*H*_3_) ppm. ^13^C-NMR (100 MHz, CDCl_3_) (Figure S6): δ = 134.8, 117.1, 99.8, 78.9, 72.4–72.0, 70.1, 65.1–64.8, 60.9, 59.1, 19.9, 15.4 ppm. The SEC trace is shown in Figure S7.

### 2.6. Synthesis of P(tBGE)_0.8_-b-P(AGE)_0.2_ 2

The P(*t*BGE)_0.8_-*b*-P(AGE)_0.2_ prepolymer was prepared analogue to the previously described procedure using caesium hydroxide (0.195 g, 1.15 mmol, 1/25 eq.), triethylene glycol monomethylether (0.189 g, 1.15 mmol, 1/25 eq.), *t*BGE (3.019 g, 23.19 mmol, 0.8 eq.) and AGE (0.629 g, 5.51 mmol, 0.2). Yield 3.11 g (85%). ^1^H-NMR (400 MHz, CDCl_3_) (Figure S8): δ = 5.87 (m, 1H, –OCH_2_C*H*CH_2_), 5.27–5.13 (dd, 2H, –OCH_2_CHC*H*_2_), 3.98 (d, 2H, –OC*H*_2_CHCH_2_), 3.64–3.38 (m, 10 H, –C*H*_2_CHO–_(backbone)_, –OCH_2_C*H*O–_(backbone)_, –O–CH_2_CH_(backbone)_C*H*_2_O–), 3.37 (s, 3H, –O–C*H*_3__(Initiator)_), 1.16 (s, 9H, –O–C(C*H*_3_)_3_) ppm. ^13^C-NMR (100 MHz, CDCl_3_) (Figure S9): δ = 134.9, 116.7, 79.3–79.0, 72.7, 72.2, 71.9, 70.8—70.1, 62.0, 59.0, 27.5 ppm. The SEC trace is shown in Figure S10.

### 2.7. Synthesis of Polyglycidol Based Microgels with in Situ Entrapment of CaLB

The procedure for the synthesis of polyglycidol based microgels was reported in detail by our group [26,27] and is complemented by the addition of CaLB here. Exemplarily the procedure for the preparation of MG **1** is explained. P(EEGE)_0.8_-*b*-P(AGE)_0.2_ **1** (1 g, 1.428 mmol allyl groups, 1 eq.), free CaLB (10 mg), 2,2′-(ethylenedioxy)diethanethiol (0.143 g, 0.785 mmol., 0.55 eq.), hexadecane (0.082 g) and 2,2-dimethoxy-2-phenylacetophenone (0.036 g) were dissolved in toluene (3.48 g). A solution of sodium dodecylsulfate (0.005 g) in water (16 g) was filtered and added to the organic phase. After a miniemulsion was prepared with a Branson Ultrasonifier 450 for 15 min (output control 3, duty cycle 50%) the emulsion was irradiated with a UV-LED cube (λ = 365 nm) for 2 h under stirring with a mechanical stirrer (1000 rpm). The microgel was purified by dialysis against water (molecular weight cutoff: 100 kDa). The enzyme concentration in the microgel was determined by amino acid analysis and measurements of the hydrolytic activity before and after dialysis.

The MG **2** as well as the CaLB free microgels MG **1.1** and MG **2.1** were prepared analogue to the previously described procedure (Tables S9 and S10).

### 2.8. Enzymatic Polymerization of ε-CL in Toluene with MG_tol_ 2

MG 2 was transferred to toluene by dialysis first against THF and afterwards against toluene. In a 25 mL flame dried Schlenk tube MG_tol_ **2** (2.63 g, water content: 530 ppm) was mixed with anhydrous toluene (0.56 g, water content: 12 ppm) and ε-CL monomer (0.83 g, water content: 39 ppm). The solution was stirred for 46 h at 50 °C. The solvent was evaporated and the product was dried in vacuum. The product was analyzed by NMR and SEC without further purification. The SEC trace and the ^1^H-NMR spectrum are shown in Figure S11.

A reference experiment with the enzyme free MG **2.1** was performed analogue to the previously described procedure (Figure S12).

## 3. Results and Discussion

**Scheme 1:**
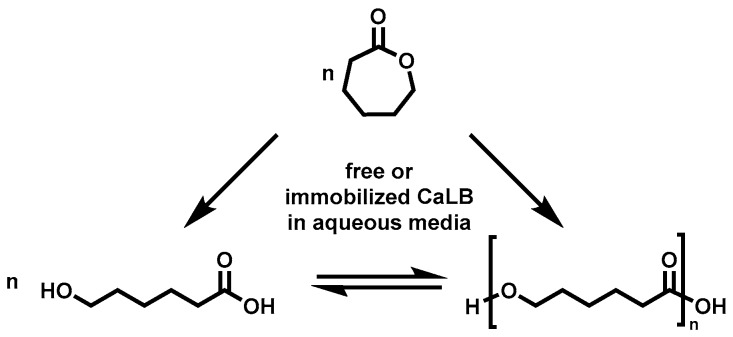
Conversion of ε-CL to 6-hydroxy caproic acid by hydrolysis or to oligo(caprolactone) by esterification catalyzed by free or immobilized CaLB in aqueous media.

ε-Caprolactone is converted in aqueous media in the presence of free or immobilized CaLB as catalyst either by hydrolysis or by ring opening esterification to form 6-hydroxy caproic acid and/or corresponding oligomers (Scheme 1). In the publication in hand we investigate the influence of the microenvironment of the lipase on the hydrolysis-esterification balance by immobilization.

Prior to the immobilization of CaLB in compartmentalized microgels by physical entrapment and the investigation of its effect on the catalytic activity, we will describe the influence of different parameters on the conversion of ε-CL: (i) The partition coefficient of ε-CL in a toluene/water mixture is determined to proof the accessibility of the monomer in hydrophobic domains, which are provided by the microgel to enable esterification; (ii) The influence of the ε-CL concentration for the conversion with free CaLB in toluene is investigated and compared to water as reaction medium; (iii) The effect of increasing water content in the hydrophobic phase will be described by performing the polymerization of ε-CL in toluene with varying water concentrations.

### 3.1. Distribution of ε-CL in a Hydrophilic/Hydrophobic Environment

**Figure 1:**
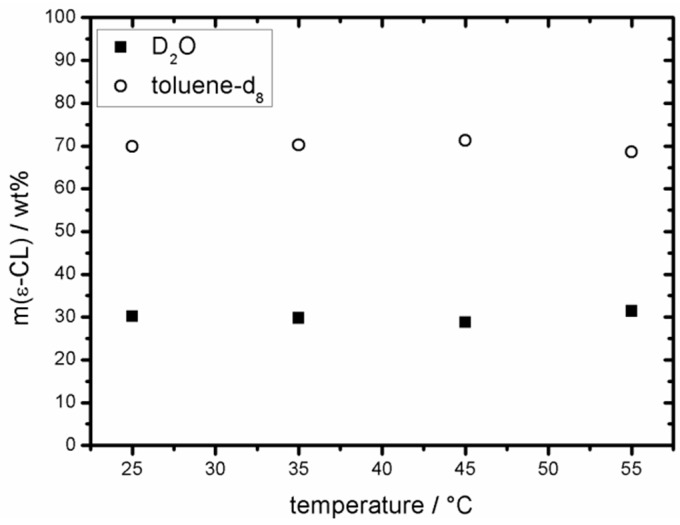
Distribution of ε-CL in toluene-d_8_ and D_2_O measured by ^1^H-NMR spectroscopy at temperatures from 25 to 55 °C.

For increasing the esterification ability of CaLB it is required to ensure that the catalysis takes place in or at the interface of a hydrophobic environment, where the monomer has to be present to a sufficient part in the hydrophobic domain. Imitating the hydrophilic/hydrophobic domains in an amphiphilic microgel we determined the distribution of ε-CL between toluene-d_8_ and D_2_O at certain temperatures by ^1^H-NMR spectroscopy with dioxane as a standard (see also Table S1). Figure 1 shows a portion of ~70 wt % ε-CL in the toluene-d_8_ phase, which stays constant over a temperature range from 25 to 55 °C. In analogy to the common log*P*_o,w_ value as a description of the average partition coefficient here the log*P*_Tol,D2O_ is determined by Equation (1) and we obtained a log *P*_Tol,D2O_ of 0.37 for ε-CL.

(1) $$\log\;P_{\mathrm{Tol},D_{2}O}=log\frac{c_{\mathrm{Tol}}^{\varepsilon -\mathrm{CL}}}{c_{D_{2}O}^{\varepsilon -\mathrm{CL}}}$$

The value lower than 1 shows the lipophilic behavior of the ε-CL. Since the polymerization reaction is perfomed within the observed temperature range at 50 °C a sufficient monomer concentration in a toluene like hydrophobic microgel is ensured.

### 3.2. Effect of the ε-CL Concentration on the Esterification Ability of Non-Immobilized CaLB

The esterification ability of non-immobilized (free) CaLB as a function of the monomer concentration *c(*ε*-CL)* was investigated both in water and in toluene. Using ε-CL concentrations from 20 wt % up to bulk conditions the products were analyzed in terms of conversion to oligomers *C*_oligo_ (fraction of ε-CL repeating units) by ^1^H-NMR and their number average molecular weight *M*_n_ by SEC (Table 1). The ^1^H-NMR spectra and SEC traces as well as the calculation of *C*_oligo_ are shown in Section 2 in the supplementary information. Important to note is, that the monomer and the toluene were not anhydrous in these experiments and contained 1530 and 266 ppm water respectively.

**Figure 2:**
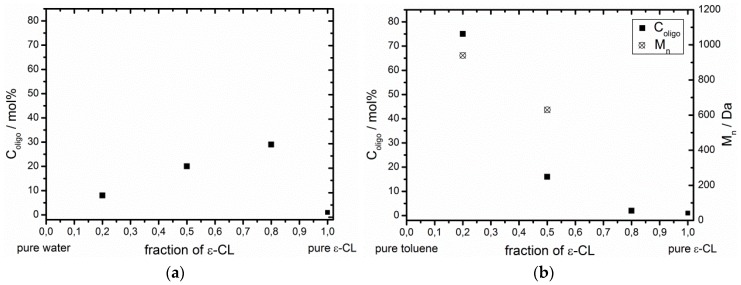
Conversion to oligomeric products *C_oligo_* determined by ^1^H-NMR spectroscopy and *M*_n_ values by SEC for the CaLB catalyzed polyesterification of ε-CL in (**a**) water and (**b**) toluene with varying ε-CL concentrations (water content ε-CL: 1530 ppm, toluene: 266 ppm).

First, regarding the esterification in water (Figure 2a), the conversion of ε-CL to oligomers for all monomer concentrations is rather low and can only be observed in the ^1^H-NMR spectrum. With increasing *c(*ε*-CL)* from 20 to 80 wt % and therefore simultaneously decreasing the water content in the reaction, the fraction of ε-CL repeating units increases from 8 to 29 mol %. However, performing the reaction in bulk another important parameter for the activity of enzymes comes into play. While CaLB is well soluble in water with up to 80 wt % of ε-CL, coagulation is observed in pure ε-CL. This deactivated form of CaLB does no longer catalyze any conversion of ε-CL, both in terms of hydrolysis and esterification. Compared to the reaction in water, in toluene an inverse trend is found (Figure 2b). At low *c(*ε*-CL)* a high conversion to oligomers with a fraction of ε-CL repeating units about 75 mol % with *M*_n_ 940 Da is observed. This is explained by the low water concentration leading to less hydrolytic activity. However, with increasing *c(*ε*-CL)* also the water content in the reaction increases, due to its presence in the monomer (1530 ppm). This results in a huge decrease of both *C*_oligo_ and *M*_n_. These investigations of a maximum esterification ability of free CaLB at around 20 wt % ε-CL are in good agreement with the investigations of Gross et al. They observed the highest conversions and molecular weights for Novozym^®^ 435 catalyzed polyesterifications in dry toluene at 30 wt % ε-CL [31]. The shift of the maximum is ascribed to effects of immobilization, e.g., it is expected, that the coagulation of the lipase is prevented by immobilization. This issue will be discussed later (Section 3.3) in this publication.

In this section, we showed the inverse dependency of the monomer concentration in water and toluene to the esterification ability of free CaLB. The observed trends are less dependent to the concentration of the monomer itself; rather more important is the strong influence of the enzyme solubility and the water content in the direct environment of the monomer and the enzyme. In toluene with a water content of 530 ppm the highest degree of esterification is observed resulting in oligo(caprolactone) with a molecular weight of 940 Da.

### 3.3. Acceptable Water Concentration in the Hydrophobic Domain

**Figure 3:**
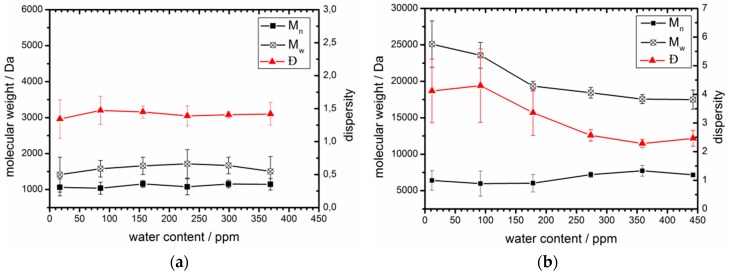
Enzymatic polymerization of ε-CL with (**a**) free CaLB and (**b**) Novozym^®^ 435 in toluene with varying water content from 0 to 400 ppm. Illustration of the progression of *M*_n_, *M*_w_ and *Ð* with increasing water content.

Detailed investigations for the optimal water concentration in the hydrophobic domain were performed by studying the esterification of ε-CL in toluene with varying water content. Considering the former results, the chosen ε-CL concentration was 20 wt % for all experiments and the free CaLB and Novozym^®^ 435 were used as catalysts. The average of triple determination of the molecular weights and the dispersity *Ð* of P(ε-CL) are shown in Table 2; the results for each particular experiment are summarized in Tables S3–S8. In Figure 3 the obtained molecular weights and dispersities are plotted against the water content for both free CaLB and Novozym^®^ 435. As it was expected, for Novozym^®^ 435 the obtained molecular weigths were in general much higher (up to *M*_n_ 7700 Da) than for the free CaLB (up to *M*_n_ 1160 Da). While the degree of polymerization stays almost constant for free CaLB, for Novozym^®^ 435 an interesting trend with increasing water content is found. On the one hand the number average molar mass increases with increasing water content from 6000 Da to 7700 Da, while on the other hand the mass average molar mass decreases starting at 25,000 Da at low water content and becoming 17,500 Da at 440 ppm water. In consequence, the dispersity decreases from 4 and converges to 2.

**Figure 4:**
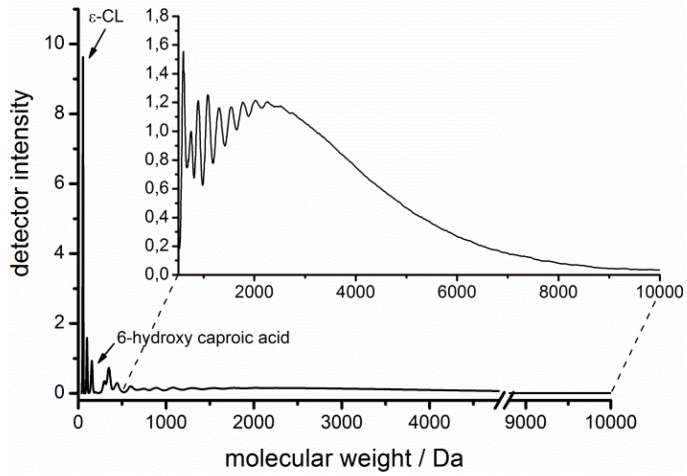
SEC trace for the polymerization of ε-CL in toluene with free CaLB.

Two exemplary molecular weight distributions of the P(ε-CL) obtained with free CaLB and Novozym^®^ 435 are analyzed. The SEC trace of the polymerization with free CaLB and a water content of 263 ppm is shown in Figure 4 (also Table S5, No. 4). It shows a high intensity signal for ε-CL (77%), a small signal for the 6-hydroxycaproic acid (6%) and an oligomer distribution with *M*_n_ 1390 Da and *Ð* 1.5 (17%). In accord with the SEC data, the ^1^H-NMR analysis shows a conversion to polymeric products of only 19 mol % demonstrating the low conversion with free CaLB.

**Figure 5:**
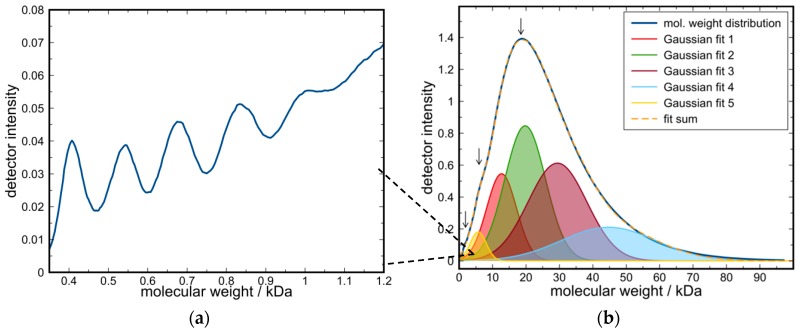
SEC trace for the polymerization of ε-CL with Novozym^®^ 435 in toluene showing (**a**) the oligomeric fraction; and (**b**) the high molecular weight fraction including the deconvolution of the original chromatogram by Gaussian fits.

In comparison, with Novozym^®^ 435 a conversion to polymer of >90 mol % at the equivalent reaction time of 46 h is obtained, showing the tremendous influence of immobilization on the reactivity and catalytic activity of CaLB. Regarding the SEC trace of the polymerization with Novozym^®^ 435 and a water content of 355 ppm (Table S8, No. 5), a multimodal distribution is observed showing both oligomeric and predominantly polymeric products leading to *M*_n_ 7800 Da and *Ð* 2.3 (Figure 5, blue line). On closer examination next to the maximum in the overall distribution at 19 kDa, two shoulders at molecular weights of 1.7 and 6.5 kDa are observed and further a small proportion of high molecular weight products >40 kDa is found. The overlay of different molecular weight distributions provides an indication for different active sites, i.e., enzymes in different active states. Deconvolution of the original spectrum using MagicPlotPro 2.7 (trial version), neglecting the oligomers, shows, that it can be fitted by exemplarily 5 different Gaussian curves (marked in Figure 5b in yellow, red, green, purple and light blue). The resulting fit sum (orange dashed line) agrees with the experimental SEC trace (adjusted *R^2^* value of 0.9996; report of fitting in the supplementary materials, Section 9).

To our best knowledge this multimodal weight distribution has never been reported so far, but from our point of view this is a very important information for the better understanding of the good performance of Novozym^®^ 435. We suggest that both the described *Ð* values > 2 as well as the multimodal distributions are ascribed to the different local environments of CaLB in Novozym^®^ 435 under the present reaction conditions, resulting in different activities and probabilities for catalysis of esterification or hydrolysis. Fernández-Lafuente et al. explain in their review [28] some of the reasons for the improvements of enzymatic activity, specificity or selectivity by immobilization and scrutinize the comparability of immobilized and free enzymes. One of the main aspects of immobilization is the prevention of aggregation, which can be found for the free enzyme. Further (covalent) immobilization rigidifies the enzymes structure preventing conformational changes by external influences or prevents the deactivation by inhibitors. On the other hand immobilization on a solid support leads to diffusional limitations resulting in e.g., a decreased substrate accessibility or internal pH gradients. Since immobilization is rather uncontrolled, it is not possible to guarantee the same local environment for all enzyme molecules on a supporting material and therefore enzymes of different activities can be found on the support like the macroporous acrylic resin of Novozym^®^ 435. Zhao and Song identified 17 trace compounds leaking from Novozym^®^ 435 into organic solvents and ionic liquids which may affect the enzymatic activity of the lipase by behaving as surfactants or inhibitors [32]. It is also worth to mention, that not only trace compounds leak from the acrylic resin, but also the physically adsorbed CaLB tends to leak from the supporting material leading to a small amount of free CaLB [33,34], which may be responsible for the low molecular weight oligomer distribution illustrated in Figure 5a. Another important factor is the interaction of the substrate ε-CL or P(ε-CL) respectively with the hydrophobic acrylic resin. It is assumed, that the growing hydrophobic polymer chain interacts more with the supporting material than the monomer or oligomers do. Further due to the increasing length the mobility of the polymer decreases and the diffusion in the supporting material is limited. Therefore longer chains are closer to the enzyme on the supporting material and continue growing, resulting in a small amount of high molecular weight polymers visible in the distribution (light blue Gaussian fit 4).

### 3.4. Preparation of Polyglycidol Based Microgels and Immobilization of CaLB

**Scheme 2:**
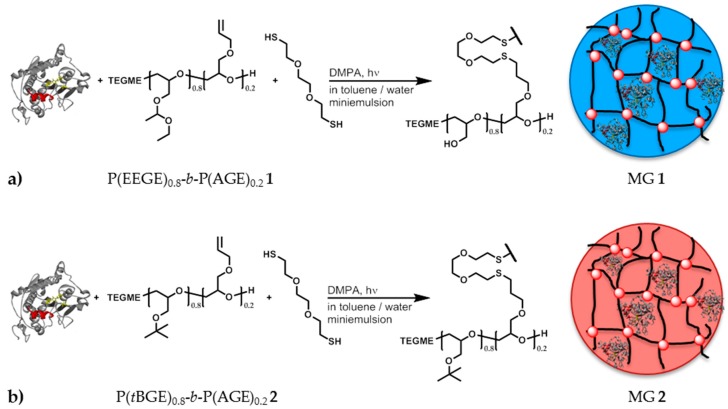
Synthetic route for the preparation of (**a**) hydrophilic microgels with hydrophobic domains using P(EEGE)_0.8_-*b*-P(AGE)_0.2_ **1** as prepolymer; and (**b**) hydrophobic microgels using P(*t*BGE)_0.8_-*b*-P(AGE)_0.2_ **2**. Free CaLB was entrapped in situ during the microgel synthesis.

In the literature, a multitude of methods and materials for enzyme immobilization are described [35,36]. Immobilization by physical adsorption, covalent or ionic binding as well as entrapping on supports from inorganic substances over biopolymers to organic polymers were reported in recent years [37,38]. However, the most suitable method for immobilization depends on both the structure of the enzyme and the type of support. In our case the preparation of the microgel in miniemulsion by a prepolymer approach in the way we published recently [26,27] opens the possibility for in situ physical entrapment of CaLB in the crosslinked polymer network (see Scheme 2). Therefore the polyglycidol prepolymer was prepared by sequential anionic polymerization of either **1** ethoxyethyl glycidyl ether (EEGE) or **2** *tert*-butylglycidyl ether (tBGE) with allylglycidyl ether (AGE) with a molar ratio of 4:1 using caesium hydroxide and triethylene glycole monomethylether (TEGME) as initiating system based on the publication by Frey et al. [39]. The prepolymers P(EEGE)_0.8_-*b*-P(AGE)_0.2_ **1** and P(tBGE)_0.8_-*b*-P(AGE)_0.2_ **2** were obtained with *M*_n_ 3600 Da (*Ð* 1.1) and 2800 Da (*Ð* 1.1) respectively (Figures S5–S10).

For the preparation of the CaLB loaded microgels MG **1** and MG **2** the respective prepolymer **1** or **2** and the CaLB were mixed in toluene with hexadecane as ultrahydrophobe, 2,2-dimethoxy-2-phenylacetophenone (DMPA) as photoinitiator and 2,2′-(ethylenedioxy) diethanethiol as crosslinker. Using sodium dodecylsulfate (SDS) as surfactant an o/w-miniemulsion of the toluene phase was prepared by ultrasonication. Irradiation of the miniemulsion with UV-light induces the thiol-ene reaction of the dithiol and the allyl moieties of the AGE building block forming a cross-linked network in which the CaLB is entrapped. While the ethoxyethyl acetal protecting group is cleaved in situ during the microgel preparation, the *tert*-butyl group stays stable under these conditions. Thus, two microgels with different compartmentalizations are formed: a hydrophilic microgel with hydrophobic domains MG **1** and an overall hydrophobic microgel MG **2** (Scheme 2). Both types of microgel were also prepared without CaLB as a reference (MG **1.1** and MG **2.1**).

The CaLB concentration in MG **1** and **2** and in Novozym^®^ 435 was determined by amino acid analysis. To avoid including not entrapped CaLB into this analytics, the microgels were dialysed against water before. Not only the free CaLB but simultaneously also low molecular weight components, which were used in the microgel preparation (e.g., SDS and DMPA), were removed in this washing step. Table 3 shows the average values of a triplicate determination of the loading as well as the theoretical enzyme concentration.

Even though this method shows a rather broad error margin, two important conclusions can be drawn from the results: (i) It was successful to immobilize CaLB into MG **1** and MG **2** by physical entrapment independent of the hydrophilic or hydrophobic microgel properties. Both measured averaged lipase concentrations correspond with the theoretical loading with a deviation of 7%; (ii) Even after threefold dialysis only a small amount of lipase is washed out, whereas the loss is more distinct for the hydrophobic than for the amphiphilic microgel. This different behavior is explained by the variation of the compartmentalization. In MG **1** interfaces between the hydrophilic and the hydrophobic domains are provided where the lipase adsorbs. Since in the overall hydrophobic MG **2** no interfaces are provided, no additional physical attachment occurs. By reengineering the CaLB making it more hydrophobic or using surfactants to increase the interaction of the lipase and the hydrophobic polymer, the problem of enzyme leakage will be overcome in the future. At this point, an important remark to the high error margin of the amino acid analysis has to be done. It is not only the result of systematic errors during the sophisticated sample preparation, but the high deviation of the single measurements rather shows the inhomogeneity of immobilization in general. As described in Section 3.3 it is nearly impossible to load a supporting material with enzyme where firstly a consistent environment is obtained and secondly the homogeneity of the enzyme distribution on or in the support can be controlled.

### 3.5. Effect of Immobilization on the Hydrolytic and Esterification Activity of CaLB

**Figure 6:**
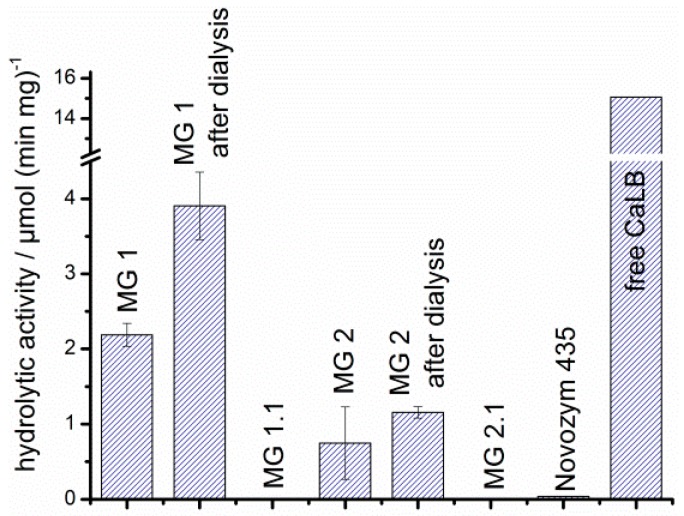
Hydrolytic activity of the CaLB loaded microgels MG **1** and MG **2** before and after dialysis (MWCO 100 kDa) and the CaLB-free microgels MG **1.1** and MG **2.1** as well as the non-immobilized CaLB as reference.

The hydrolytic activities of free and the immobilized CaLB were analyzed by *p*NPB assay and are shown in Figure 6. For the immobilized CaLB either in a microgel or adsorbed on the acrylic resin in Novozym^®^ 435 a strong decrease compared to the free CaLB is determined. While the free CaLB has a hydrolytic activity of 15.1 µmol(min·mg)^−1^ it is reduced in the MG **1** to 3.9 µmol(min·mg)^−1^. Interestingly, the reduction of the hydrolytic activity is much more intense for the hydrophobic MG **2** (1.2 µmol(min·mg)^−1^) and even more for Novozym^®^ 435 (0.04 µmol(min·mg)^−1^). Hence, we conclude a correlation between the hydrolytic activity and the hydrophobicity of the supporting material. Additionally, comparing the hydrolytic activities of each microgel before and after dialysis a slightly higher activity after the dialysis is observed in both cases. This is explained by the removal of low molecular weight compounds during the dialysis. SDS, unreacted cross-linker or not cross-linked polymer chains can act as surfactant or inhibitor and therefore decrease the activity of the CaLB. The decrease of hydrolytic activity might be explained either by deactivation of the enzyme or by retardation of the hydrolysis as the local water content in the environment of the lipase is lowered significantly by immobilization. Hence, the question arises whether the decreased activities, which we observed for CaLB entrapped in the amphiphilic MG **1** as well as in the hydrophobic MG **2** will be connected to an increased esterification/polymerization activity.

The esterification ability for the MG **2** and MG **2.1**, was investigated for a polymerization of ε-CL in toluene and compared to the identical experiments using free CaLB or Novozym^®^ 435 presented in Section 3.3. Therefore, the solvent of the microgels was substituted by dialysis from water to toluene, hereinafter referred to as MG_tol_ **2** and MG_tol_ **2.1** respectively. Since the volume was held constant before and after dialysis, the CaLB concentration in MG_tol_ **2** is also assumed to be constant with an enzyme loading of 0.34 mg·mL^−1^. Performing the polymerization of ε-CL with 1 mg CaLB (2.94 mL MG_tol_ **2**) a molecular weight for P(ε-CL) of *M*_n_ 4900 Da (*M*_w_ 11,700 Da) and a conversion to the polymeric product of 87 mol % was obtained (Table 4, Figure S11). Compared to free CaLB, we observed a huge increase in the conversion to polymer as well as a four times higher molecular weight. While the hydrolytic activity of Novozym^®^ 435 was the lowest, the results show that its polymerization ability is even higher than for the MG_tol_ **2**. Since the reference experiment using enzyme free MG_tol_ **2.1** does not lead to any kind of conversion we proved that the microgel itself has no influence on the initiation of the polymerization (see Table 4, Figure S12).

These results do not only show that the decrease in hydrolytic activity is not caused by denaturation, but rather that it is an effect of the hydrophobicity of the microenvironment of the supporting material. With increasing hydrophobicity of the supporting material the hydrolytic activity of CaLB is decreased, while simultaneously the polymerization activity is increased.

## 4. Conclusions

In the presented study, we described the successful immobilization of *Candida antarctica* lipase B in poly(glycidol) based compartmentalized microgels with varying polarity and investigated the influence of immobilization on the hydrolytic and esterification activity of CaLB. Therefore, we first proved the good accessibility of ε-CL into toluene as a model, imitating the hydrophobic domain in a microgel ($c_{\mathrm{Tol}}^{\varepsilon -\mathrm{CL}}=70\%$, *logP*_Tol/H2O_ = 0.37). Further, we investigated that not the monomer concentration, but rather the solubility of CaLB and the water concentration in the hydrophobic medium, which should not exceed 500 ppm, play the most important role for the esterification ability. Comparing the polymerization of ε-CL catalyzed by free CaLB and by Novozym^®^ 435 in toluene with varying water content, we observed a constant degree of polymerization for free CaLB, while the immobilized form in Novozym^®^ 435 gave up to 7 times higher molecular weights. Taking a closer look to the polymerization products for Novozym^®^ 435 we observed a multimodal molecular weight distribution and a decreasing dispersity converging from 4 to 2 with increasing water content. These observations are ascribed to the coexistence of CaLB in different local environments on the acrylic resin leading to different polymerization activities. Finally, we prepared amphiphilic and hydrophobic microgels and immobilized CaLB by physical entrapment, which represents an elegant immobilization procedure, since the lipase is entrapped in situ during the microgel preparation. The immobilization was successful in terms of loading efficiency and preservation of enzymatic activity. In both microgels, as well as in Novozym^®^ 435, the hydrolytic activity was decreased compared to free CaLB. The intensity of reduction is hereby related to the hydrophilic-lipophilic balance within the microgel, changing the local water content in the close environment of the lipase and retarding the hydrolysis of the substrate. For MG_tol_ **2** and Novozym^®^ 435 we could prove that the reduced hydrolytic activity is simultaneously connected to a strong improvement in esterification ability. Thus, in this work, we have presented an elegant way for CaLB immobilization in microgels and demonstrated a huge effect of the hydrophobicity of the microenvironment of CaLB on the catalytic performance of the lipase.

## Figures and Tables

**Table 1:** CaLB catalyzed ROP of ε-CL in bulk, water and toluene with varying ε-CL concentrations; conversion to oligomers and their molecular weights.

Solvent	Fraction of ε-CL	Fraction of solvent	H_2_O Content/ppm	*C*_oligo_/mol % ^(1)^	*M*_n_/Da ^(2)^
bulk ε-CL	1	0	1,530	1	- ^(3)^
H_2_O	0.8	0.2	-	29	- ^(3)^
H_2_O	0.5	0.5	-	20	- ^(3)^
H_2_O	0.2	0.8	-	8	- ^(3)^
toluene	0.8	0.2	1,280	2	- ^(3)^
toluene	0.5	0.5	900	16	630
toluene	0.2	0.8	530	75	940

^(1)^ fraction of ε-CL repeating units determined by ^1^H-NMR spectroscopy in CDCl_3_; ^(2)^ SEC in THF; ^(3)^ low intensity signal (<600 Da) beyond the calibration range.

**Table 2:** Enzymatic ROP of ε-CL in toluene with varying water content using free CaLB and Novozym^®^ 435 as catalyst ^(1)^; results of SEC analyses.

Toluene (Dry)/%	Toluene (water saturated)/%	Experiments with free CaLB			Experiments with Novozym^®^ 435		
		Total H_2_O content/ppm ^(2)^	*M*_n_/Da ^(3)^	*Ð* ^(3)^	Total H_2_O content/ppm ^(4)^	*M*_n_/Da ^(3)^	*Ð* ^(3)^
100	0	17	1,090	1.3	12	6,400	4.1
80	20	85	1,060	1.5	92	6,000	4.3
60	40	156	1,160	1.5	178	6,000	3.4
40	60	230	1,080	1.4	274	7,200	2.6
20	80	299	1,160	1.4	359	7,700	2.3
0	100	369	1,140	1.4	442	7,200	2.5

^(1)^ For experimental details see Tables S3–S8; ^(2)^ water content in reactants: dry toluene: 16 ppm, sat. toluene: 520 ppm, ε-CL: 20 ppm; ^(3)^ SEC in THF (average of triple determination); ^(4)^ water content in reactants: dry toluene: 5 ppm, sat. toluene: 547 ppm, ε-CL: 26 ppm.

**Table 3:** Loading efficiency of MG **1** and MG **2** before and after dialysis (MWCO 100 kDa) and the CaLB-free microgels MG **1.1** and MG **2.1** as well as Novozym^®^ 435 as reference.

Type of supporting material	Prepolymer	Enzyme	[CaLB]/mg·mL^−1^	
			Before dialysis ^(1)^	After dialysis ^(1)^
MG **1**	P(EEGE)-*b*-(PAGE)	CaLB	0.58 (0.54)	0.44 (0.48)
MG **1.1**	P(EEGE)-*b*-P(AGE)	-	0	0
MG **2**	P(*t*BGE)-*b*-P(AGE)	CaLB	0.54 (0.50)	0.34 (0.46)
MG **2.2**	P(*t*BGE)-*b*-P(AGE)	-	0	0
acrylic resin ^(2)^	-	CaLB	0.48 ^(3)^	

^(1)^ experimental (theoretical); ^(2)^ Novozym^®^ 435; ^(3)^ without dialysis.

**Table 4:** Enzymatic ROP of ε-CL in toluene using free and immobilized CaLB as catalyst; results of NMR and SEC analyses.

Catalyst	H_2_O content/ppm	C_oligo_/mol % ^(1)^	*M*_n_/Da ^(2)^	*Ð* ^(2)^
free CaLB	369	13	1,140	1.4
Novozym^®^ 435	359	96	7,700	2.3
MG_tol_ 2	347	87	4,900	2.4
MG_tol_ 2.1	209	0	- ^(3)^	- ^(3)^

^(1)^ fraction of ε-CL repeating units determined by ^1^H-NMR spectroscopy in CDCl_3_; ^(2)^ SEC in THF; ^(3)^ no oligomers.

## Supplementary Materials

The following are available online at www.mdpi.com/2073-4360/8/10/372/s1: Figure S1. ^1^H-NMR spectrum of ε-CL in D_2_O with dioxane standard at 25 °C (No. 1.1 in Table S1); Figure S2. ^1^H-NMR spectrum of ε-CL in toluene-*d*_8_ with dioxane standard at 25 °C (No. 1.1 in Table S1); Figure S3. Molar mass distribution for the enzymatic polymerization of ε-CL with CaLB for (a) a ratio of ε-CL and water of 1:4 (No. 2.4 in Table S2) and (b) a ratio of ε-CL and toluene 1:4 (No. 2.7 in Table S2); Figure S4. ^1^H-NMR spectrum of the polymerization product of experiment No. 2.6 in Table S2 with the signals for the ε-CL monomer (a), the hydrolysis product 6-hydroxyhexanoic acid (b) and the P(ε-CL) (c); Figures S5–S7. ^1^H-NMR and ^13^C-NMR spectra as well as SEC trace of P(EEGE)_0.8_-*b*-P(AGE)_0.2_ **1**; Figures S8–S10: ^1^H-NMR and ^13^C-NMR spectra as well as SEC trace of P(*t*BGE)_0.8_-*b*-P(AGE)_0.2_ **2**; Figure S11: (a) SEC trace and (b) ^1^H-NMR spectrum for the enzymatic polymerization of ε-CL with MG_tol_ **2**; Figure S12: (a) SEC trace and (b) ^1^H-NMR spectrum for the enzymatic polymerization of ε-CL with MG_tol_ **2.1**; Table S1: Weights of ε-CL, D_2_O, toluene-d_8_ and dioxane for different temperatures; Table S2: Weights of ε-CL, H_2_O, toluene and CaLB and the corresponding conversions to polymer *C_oligo_* and molecular weights *M*_n_ and *M*_w_ determined by ^1^H-NMR and SEC; Tables S3–S5: Enzymatic polymerization with CaLB; Tables S6–S8: Enzymatic polymerization with Novozym^®^ 435; Table S9: Weighed portions for the synthesis of P(EEGE)-*b*-P(AGE) **1** based microgels MG **1** and MG **1.1**; Table S10: Weighed portions for the synthesis of P(*t*BGE)-*b*-P(AGE) **2** based microgels MG **2** and MG **2.1**, Section 8. Cloning, production and purification of CaLB, Section 9. Report of deconvolution of the molecular weight distribution obtained with Novozym^®^ 435.

## Abbreviations

The following abbreviations are used in this manuscript:

AGE	allyl glycidyl erher
CaLB	*Candida antarctica* lipase B
DMPA	2,2-dimethoxy-2-phenylacetophenone
ε-CL	ε-caprolactone
EEGE	ethoxyethyl glycidyl ether
*Ð*	dispersity index
MBDSTFA	*N*-methyl-*N*-*tert*-butyldimethylsilyl-trifluoroacetamide
MG	microgel
MTP	microtiterplate
PDL	pentadecalactone
*p*NBP	*para*-nitrophenol butyrate
ROP	ring opening polymerization
SDS	sodium dodecylsulfate
*t*BGE	*tert*-butyl glycidyl ether
TEA	triethanolamine
TEGME	triethylene glycol monomethylether
UDL	11-undecanolide
YPD	yeast extract peptone dextrose
