# Supplementary material for: CaLB Catalyzed Conversion of ε-Caprolactone in Aqueous Medium. Part 1: Immobilization of CaLB to Microgels
Source: Polymers (Basel). 2016 Oct 19;8(10):372. doi: 10.3390/polym8100372 (PMC6432092; doi:10.3390/polym8100372)
Supplement: Supplementary file 1 [file polymers-08-00372-s001.pdf]

Supplementary Materials: CaLB Catalyzed Conversion of ε-Caprolactone in Aqueous Medium. Part 1: Immobilization of CaLB to Microgels

Stefan Engel, Heidi Höck, Marco Bocola, Helmut Keul, Ulrich Schwaneberg and Martin Möller

1. Distribution of ε-CL in a Hydrophilic/Hydrophobic Environment S2

2. Effect of the ε-CL Concentration on the Esterification Ability of Non-Immobilized CaLB S3

3. Acceptable Water Concentration in the Hydrophobic Domain S4

4. Synthesis of $P(EEGE)_{0.8}$-*b*-$P(AGE)_{0.2}$ 1 S6

5. Synthesis of $P(tBGE)_{0.8}$-*b*-$P(AGE)_{0.2}$ 2 S7

6. Synthesis of Polyglycidol Based Microgels S8

7. Enzymatic ROP of ε-CL with CaLB Immobilized in Microgels S9

8. Cloning, Production and Purification of CaLB S10

8.1. Cloning of Candida antarctica lipase B into the pGAPz Expression Vector **S10**

8.2. Expression of CaLB in 96-well Microtiter Plates **S10**

8.3. p-Nitrophenyl butyrate (pNPB) Assay in MTP Format for CaLB Activity Measurement **S10**

8.4. Production of CaLB in Shake Flask and Purification **S10**

9. Report of Deconvolution of the Molecular Weight Distribution Obtained with Novozym® 435 S11

1. Distribution of ε-CL in a Hydrophilic/Hydrophobic Environment

Table S1. Weights of ε-CL, D_2O, toluene-d_8 and dioxane for different temperatures; distribution of ε-CL in the D_2O and toluene-d_8 phase.

No	T/°C	m(ε-CL)/g	m(D_2O)/g	m(toluene)/g	m(dioxane)/g		c(ε-CL) [1]/molL^{-1}	
					D_2O phase	toluene-d_8 phase	D_2O phase	toluene-d_8 phase
1.1	25	0.0879	1.1056	0.9342	0.0104	0.0096	0.223	0.518
1.2	35	0.0873	1.1066	0.9375	0.0077	0.0100	0.218	0.515
1.3	45	0.0901	1.1074	0.9404	0.0101	0.0099	0.221	0.547
1.4	55	0.0870	1.1063	0.9414	0.0104	0.0092	0.224	0.491

[1] determined by ^{1}H-NMR spectroscopy.

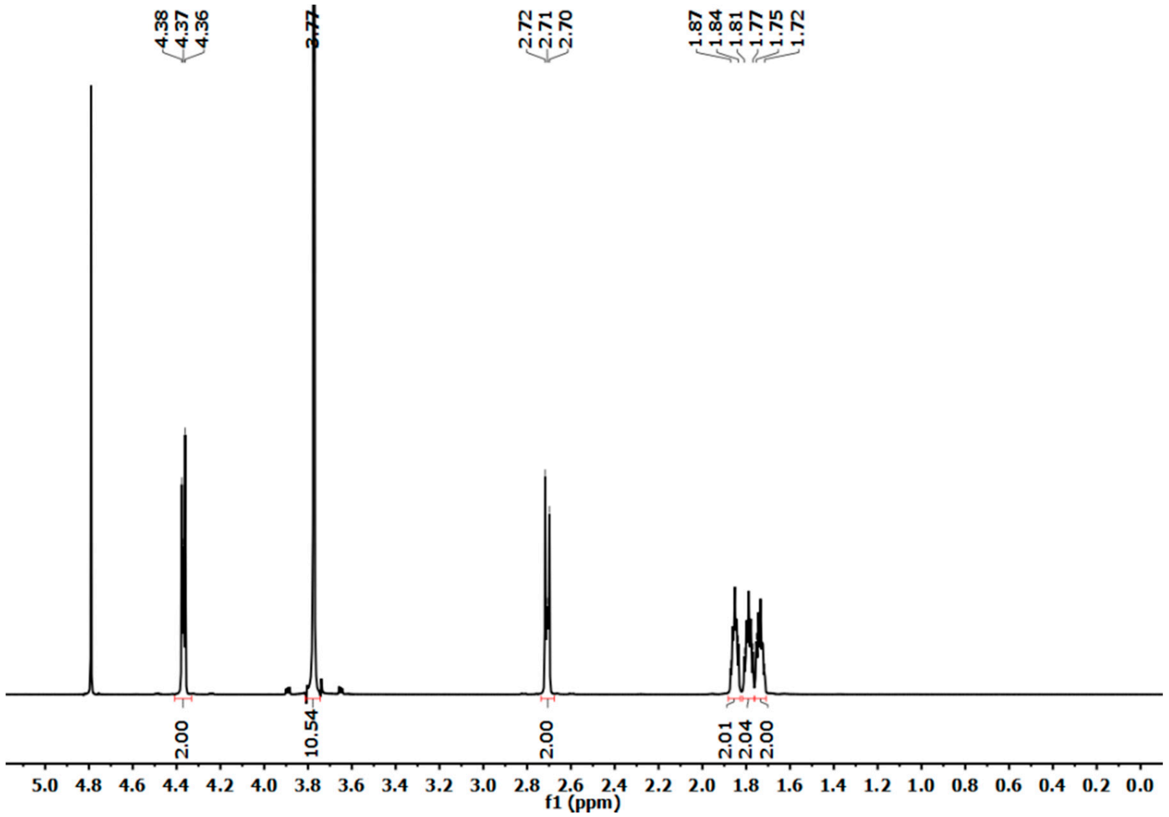

Figure S1. ^{1}H-NMR spectrum of ε-CL in D_2O with dioxane standard at 25 °C (No. 1.1 in Table S1). ^{1}H-NMR (400 MHz, D_2O): δ = 4.38–4.36 (m, 2H^a, –CH_2O–), 3.77 (s, 8H, dioxane), 2.72–2.70 (m, 2H^b, –CH_2CO–), 1.87–1.84 (m, 2H^c, –$CH_2$$CH_2$O–), 1.81–1.77 (m, 2H^d, –$CH_2$$CH_2$CO), 1.75–1.72 (m, 2H^e, –$CH_2$$CH_2$$CH_2$) ppm.

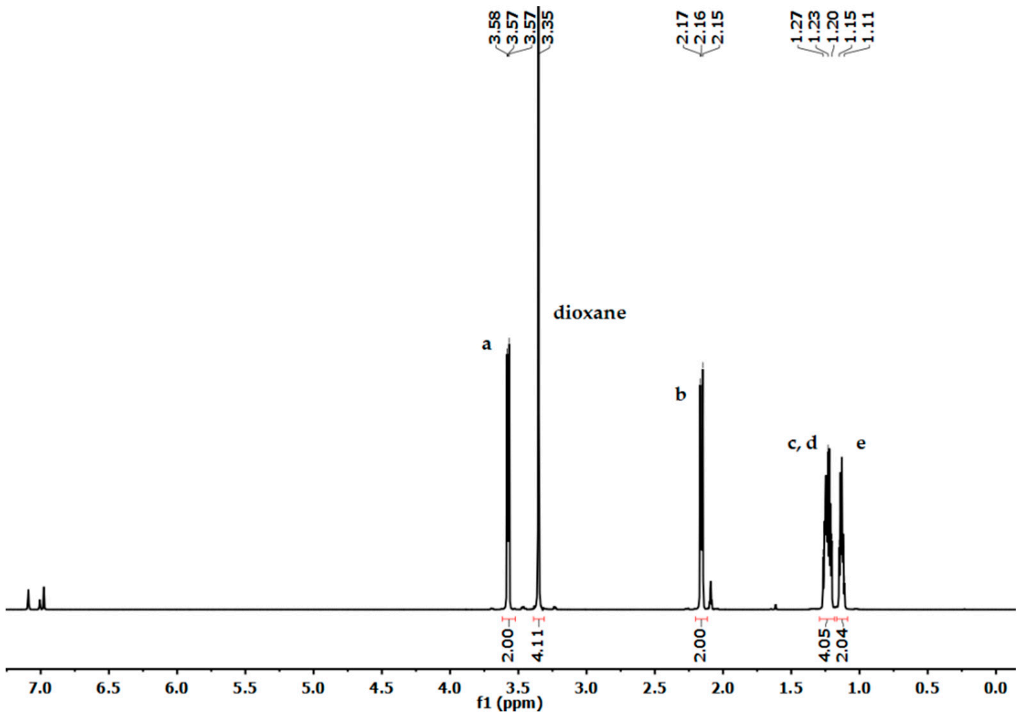

Figure S2. ^{1}H-NMR spectrum of ε-CL in toluene-d_8 with dioxane standard at 25 °C (No. 1.1 in Table S1). ^{1}H-NMR (400 MHz, toluene-d_8): δ = 3.58–3.57 (m, 2H^a, –*CH*$_2$O–), 3.35 (s, 8H, dioxane), 2.17–2.15 (m, 2H^b, –*CH*$_2$CO–), 1.27–1.23 (m, 2H^c, –*CH*$_2$CH$_2$O–), 1.23–1.20 (m, 2H^d, –*CH*$_2$CH$_2$CO), 1.15–1.11 (m, 2H^e, –CH$_2$*CH*$_2$CH$_2$) ppm.

2. Effect of the ε-CL Concentration on the Esterification Ability of Non-Immobilized CaLB

Table S2. Weights of ε-CL, H_2O, toluene and CaLB and the corresponding conversions to polymer C_{oligo} and molecular weights M_n and M_w determined by ^{1}H-NMR and SEC respectively.

No.	m(ε-CL)/g (wt %) [1]	m(H_2O)/g (wt %)	m(toluene)/g (wt %) [2]	m(CaLB)/mg	C_{oligo}/% [3]	M_n/Da [4]	M_w/Da [4]
2.1	4.01 (100)	-	-	1	1	120	120
2.2	3.20 (80)	0.80 (20)	-	1	29	120	120
2.3	2.00 (50)	2.00 (50)	-	1	20	120	120
2.4	0.80 (20)	3.20 (80)	-	1	8	120	120
2.5	3.20 (80)	-	0.80 (20)	1	2	115	120
2.6	2.01 (50)	-	2.00 (50)	1	16	115	120
2.7	0.82 (20)	-	3.20 (80)	1	75	910	1,260

[1] water content: 1530 ppm; [2] water content: 266 ppm; [3] ^{1}H-NMR in $CDCl_3$, [4] SEC in THF.

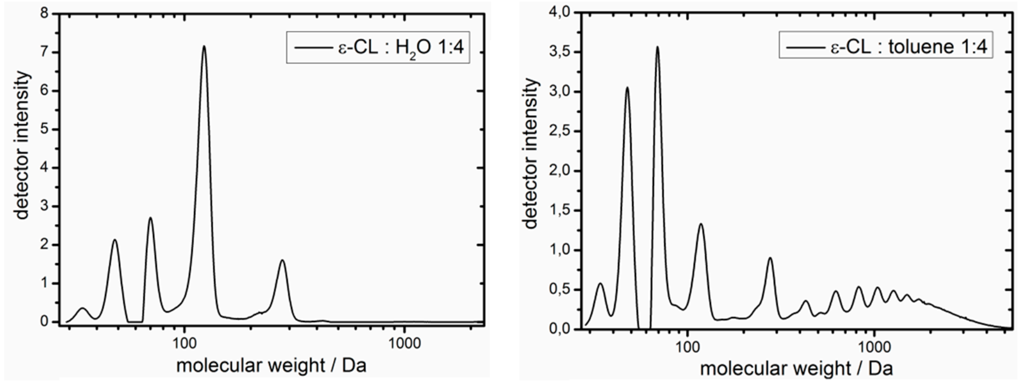

(**a**) (**b**)

Figure S3. Molar mass distribution for the enzymatic polymerization of ε-CL with CaLB for (**a**) a ratio of ε-CL and water of 1:4 (No. 2.4 in Table S2) and (**b**) a ratio of ε-CL and toluene 1:4 (No. 2.7 in Table S2).

The conversion of ε-CL to oligomers (C_{oligo}) or polymers (C_{polym}) respectively is determined from the ^{1}H-NMR spectra (Figure S4) by using the discrete signals of the protons in γ–position for ε-CL, and the respective protons of 6-hydroxyhexanoic acid and the oligomer/polymer. While the signal at δ = 4.15 ppm (1a) is assigned to the ε-CL, the signal for the polymer/oligomer is found at δ = 3.97 ppm (1c) if the spectrum is measured in $CDCl_3$. The signal of the end group (1b) of both the oligomer and the 6-hydroxyhexanoic acid is found at a shift of δ = 3.54 ppm. Therefore the conversion C_{oligo}/C_{polym} is calculated by

$$\boldsymbol{C}_{\mathbf{oligo}} = \frac{\int \mathbf{1}\boldsymbol{c}}{\int \mathbf{1}\boldsymbol{a} + \int \mathbf{1}\boldsymbol{b} + \int \mathbf{1}\boldsymbol{c}} \tag{1}$$

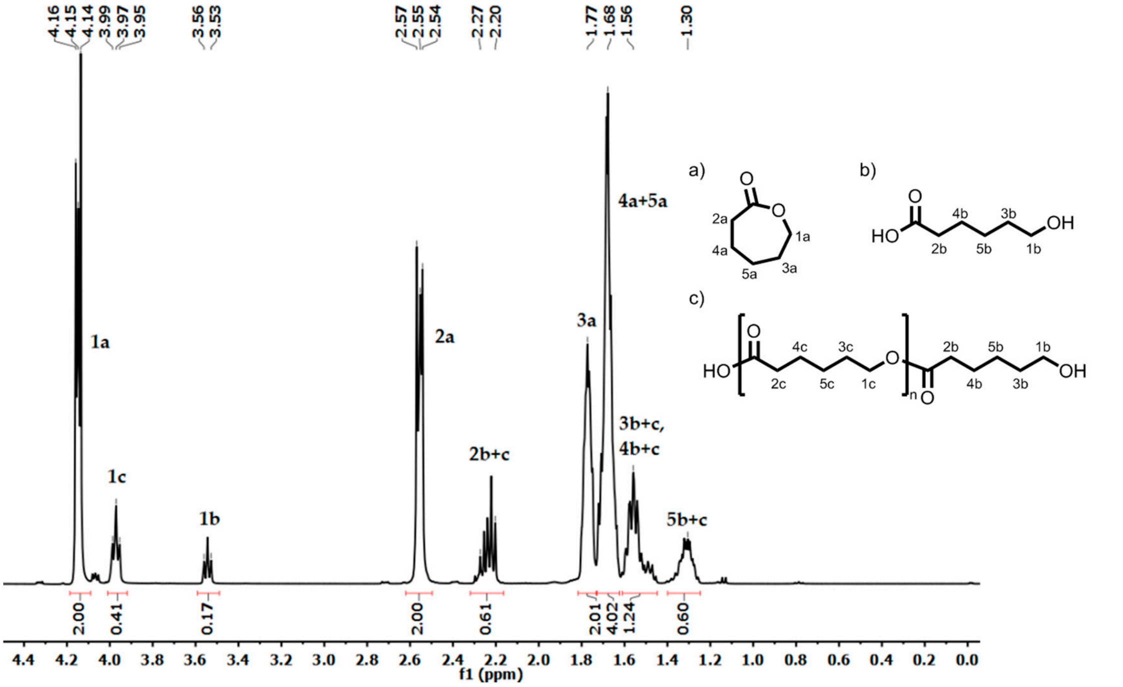

Figure S4. ^{1}H-NMR spectrum in $CDCl_3$ of the polymerization product of experiment No. 2.6 in Table S2 with the signals for the ε-CL monomer (**a**), the hydrolysis product 6-hydroxyhexanoic acid (**b**) and the poly/oligo(ε-CL) (**c**).

3. Acceptable Water Concentration in the Hydrophobic Domain

Table S3. Enzymatic polymerization with 1 mg CaLB: Weights of anhydrous toluene (6 ppm H_2O), water saturated toluene (315 ppm H_2O) and anhydrous ε-CL (65 ppm H_2O); the corresponding conversions to oligomers C_{oligo} and molecular weights M_n determined by ^{1}H-NMR and SEC respectively.

No.	Anhydr. toluene/g (%)	H_2O sat. toluene/g (%)	ε-CL/g	Total H_2O content/ppm	C_{oligo}/%	M_n/Da	Đ
1	3.26 (100)	0.00 (0)	0.82	18	7	950	1.2
2	2.67 (80)	0.62 (20)	0.85	66	4	940	1.2
3	1.94 (60)	1.27 (40)	0.81	116	10	1,100	1.5
4	1.24 (40)	1.95 (60)	0.81	168	9	930	1.2
5	0.59 (20)	2.63 (80)	0.81	221	12	1,100	1.4
6	0.00 (0)	3.23 (100)	0.81	267	15	1,200	1.6

Table S4. Enzymatic polymerization with 1 mg CaLB: Weights of anhydrous toluene (7 ppm H_2O), water saturated toluene (504 ppm H_2O) and anhydrous ε-CL (49 ppm H_2O); the corresponding conversions to oligomers C_{oligo} and molecular weights M_n determined by ^{1}H-NMR and SEC respectively.

No.	Anhydr. toluene/g (%)	H_2O sat. toluene/g (%)	ε-CL/g	Total H_2O content/ppm	C_{oligo}/%	M_n/Da	Đ
1	3.20 (100)	0.00 (0)	0.84	16	3	850	1.1
2	2.68 (80)	0.65 (20)	0.83	97	5	900	1.5
3	1.94 (60)	1.30 (40)	0.81	177	15	1,080	1.3
4	1.26 (40)	1.95 (60)	0.81	258	6	910	1.5
5	0.61 (20)	2.53 (80)	0.82	330	12	1,060	1.4
6	0.00 (0)	3.25 (100)	0.81	419	10	930	1.2

Table S5. Enzymatic polymerization with 1 mg CaLB: Weights of anhydrous toluene (16 ppm H_2O), water saturated toluene (520 ppm H_2O) and anhydrous ε-CL (20 ppm H_2O); the corresponding conversions to oligomers C_{oligo} and molecular weights M_n determined by ^{1}H-NMR and SEC respectively.

No.	Anhydr. toluene/g (%)	H_2O sat. toluene/g (%)	ε-CL/g	Total H_2O content/ppm	C_{oligo}/%	M_n/Da	Đ
1	3.21 (100)	0.00 (0)	0.82	17	11	1,470	1.8
2	2.63 (80)	0.59 (20)	0.81	92	9	1,340	1.7
3	1.94 (60)	1.25 (40)	0.80	175	9	1,310	1.5
4	1.23 (40)	1.95 (60)	0.80	263	19	1,390	1.5
5	0.59 (20)	2.60 (80)	0.83	345	15	1,310	1.5
6	0.00 (0)	3.20 (100)	0.78	420	13	1,300	1.5

Table S6. Enzymatic polymerization with 10 mg Novozym® 435 (1/10 wt/wt): Weights of anhydrous toluene (5 ppm H_2O), water saturated toluene (547 ppm H_2O) and anhydrous ε-CL (26 ppm H_2O); the corresponding conversions to polymers C_{polym} and molecular weights M_n determined by ^{1}H-NMR and SEC respectively.

No.	Anhydr. toluene/g (%)	H_2O sat. toluene/g (%)	ε-CL/g	Total H_2O content/ppm	C_{polym}/%	M_n/Da	Đ
1	3.23 (100)	0.00 (0)	0.83	10	92	4,500	5.6
2	2.60 (80)	0.63 (20)	0.80	95	94	4,900	4.8
3	1.93 (60)	1.23 (40)	0.83	176	91	6,000	3.2
4	1.25 (40)	1.93 (60)	0.83	271	95	7,500	2.5
5	0.60 (20)	2.58 (80)	0.80	359	97	6,800	2.5
6	0.00 (0)	3.22 (100)	0.81	446	97	7,200	2.5

Table S7. Enzymatic polymerization with 10 mg Novozym® 435 (1/10 wt/wt): Weights of anhydrous toluene (5 ppm H_2O), water saturated toluene (547 ppm H_2O) and anhydrous ε-CL (26 ppm H_2O); the corresponding conversions to polymers C_{polym} and molecular weights M_n determined by ^{1}H-NMR and SEC respectively.

No.	Anhydr. toluene/g (%)	H_2O sat. toluene/g (%)	ε-CL/g	Total H_2O content/ppm	C_{polym}/%	M_n /Da	Đ
1	3.19 (100)	0.00 (0)	0.82	9	90	7,400	2.9
2	2.64 (80)	0.57 (20)	0.83	87	90	8,400	2.3
3	1.98 (60)	1.25 (40)	0.81	179	91	4,600	4.4
4	1.26 (40)	1.96 (60)	0.84	275	93	7,400	2.4
5	0.59 (20)	2.60 (80)	0.80	362	97	8,600	2.1
6	0.00 (0)	3.22 (100)	0.80	446	92	7,500	2.1

Table S8. Enzymatic polymerization with 10 mg Novozym® 435 (1/10 wt/wt): Weights of anhydrous toluene (13 ppm H_2O), water saturated toluene (541 ppm H_2O) and anhydrous ε-CL (26 ppm H_2O); the corresponding conversions to polymers C_{polym} and molecular weights M_n determined by ^{1}H-NMR and SEC respectively.

No.	Anhydr. toluene/g (%)	H_2O sat. toluene/g (%)	ε-CL/g	Total H_2O content/ppm	C_{polym}/%	M_n/Da	Đ
1	3.24 (100)	0.00 (0)	0.79	16	91	7,300	3.9
2	2.62 (80)	0.58 (20)	0.84	93	91	4,600	5.6
3	1.97 (60)	1.24 (40)	0.83	180	93	7,500	2.5
4	1.26 (40)	1.96 (60)	0.83	275	92	6,700	2.8
5	0.62 (20)	2.57 (80)	0.81	355	94	7,800	2.3
6	0.00 (0)	3.18 (100)	0.81	435	92	6,800	2.8

4. Synthesis of P(EEGE)$_{0.8}$-*b*-P(AGE)$_{0.2}$ 1

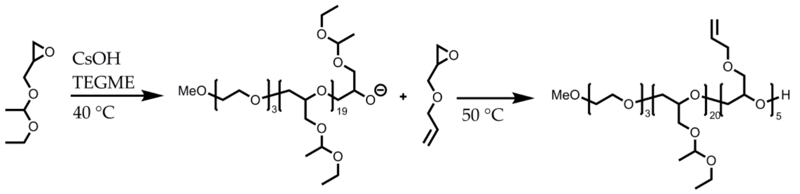

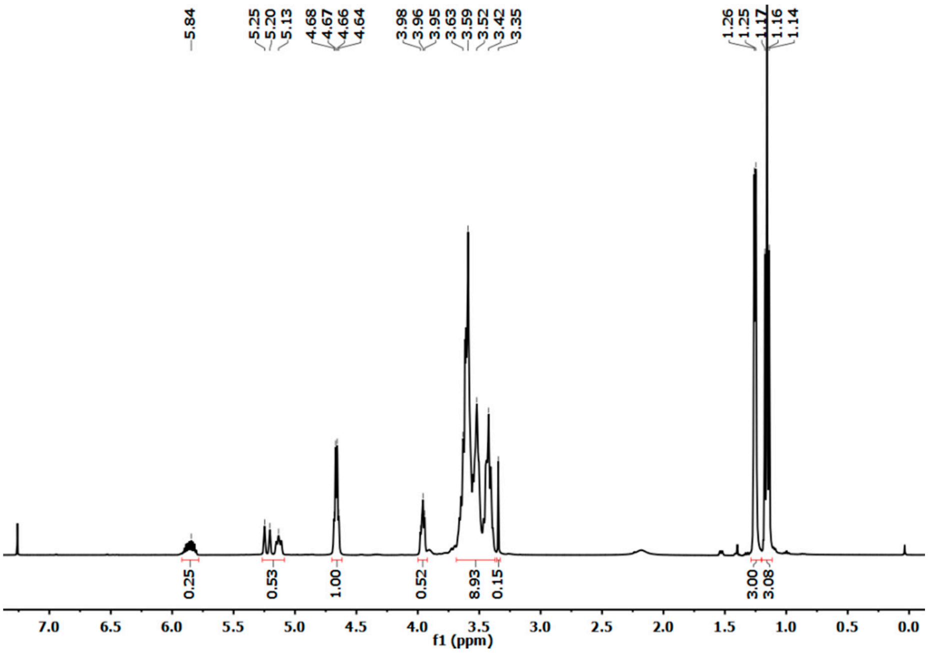

Figure S5. ^{1}H-NMR spectrum (in $CDCl_3$) of P(EEGE)$_{0.8}$-*b*-P(AGE)$_{0.2}$ 1. ^{1}H-NMR (400 MHz, $CDCl_3$): δ = 5.86 (m, 1H, –OCH_2C*H*CH_2), 5.26–5.13 (dd, 2H, –OCH_2CHC*H*$_2$), 4.68 (m, 1H, CH_3C*H*–), 3.96 (d, 2H, –OC*H*$_2CHCH_2$), 3.63–3.44 (m, 12H, –C*H*$_2$CHO-$_{(backbone)}$, –CH_2C*H*O-$_{(backbone)}$, –$OCH_2CH_{(backbone)}C$*H*$_2$O–, –OC*H*$_2CH_3$), 3.37 (s, 3H, O–C*H*$_{3(Initiator)}$), 1.28–1.27 (d, 3H, –CHC*H*$_3$), 1.18 (t, 3H, –OCH_2C*H*$_3$) ppm.

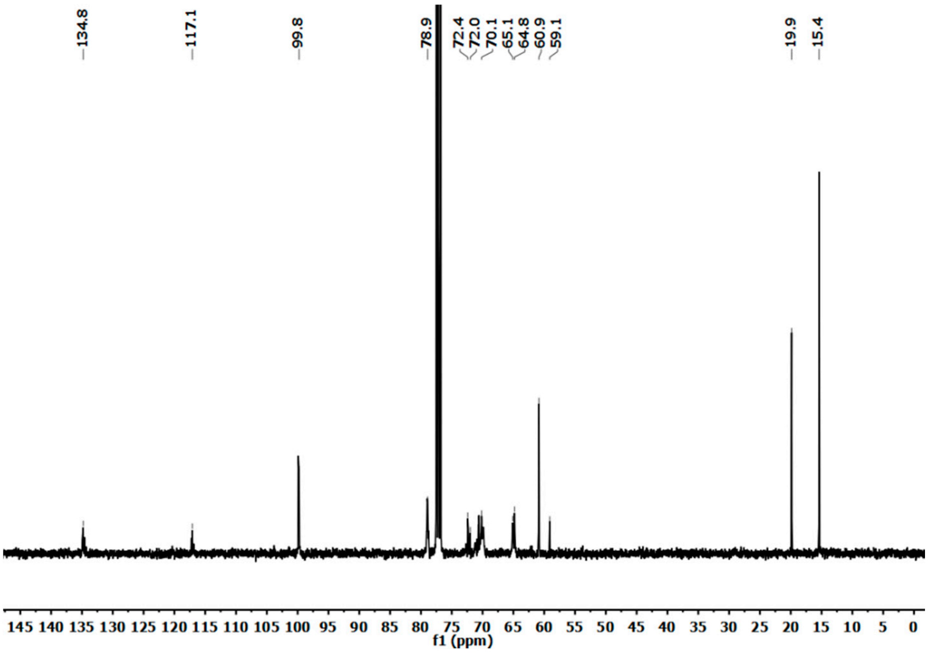

Figure S6. ^{13}C-NMR spectrum (in $CDCl_3$) of P(EEGE)$_{0.8}$-*b*-P(AGE)$_{0.2}$ 1. ^{13}C-NMR (100 MHz, $CDCl_3$): δ = 134.8, 117.1, 99.8, 78.9, 72.4–72.0, 70.1, 65.1-64.8, 60.9, 59.1, 19.9, 15.4 ppm.

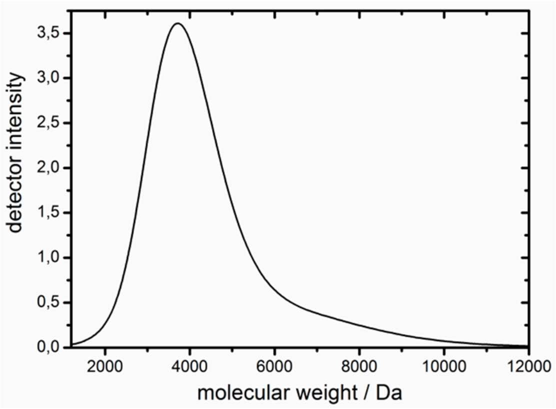

Figure S7. SEC trace (in THF) of P(EEGE)$_{0.8}$-*b*-P(AGE)$_{0.2}$ **1**.

5. Synthesis of P(*t*BGE)$_{0.8}$-*b*-P(AGE)$_{0.2}$ 2

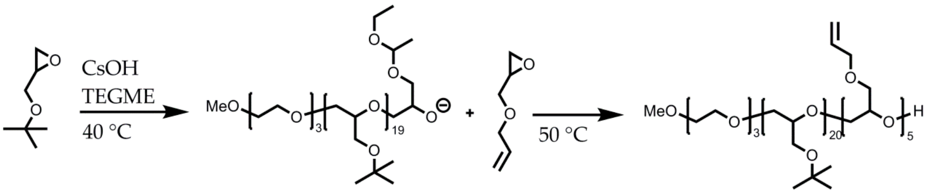

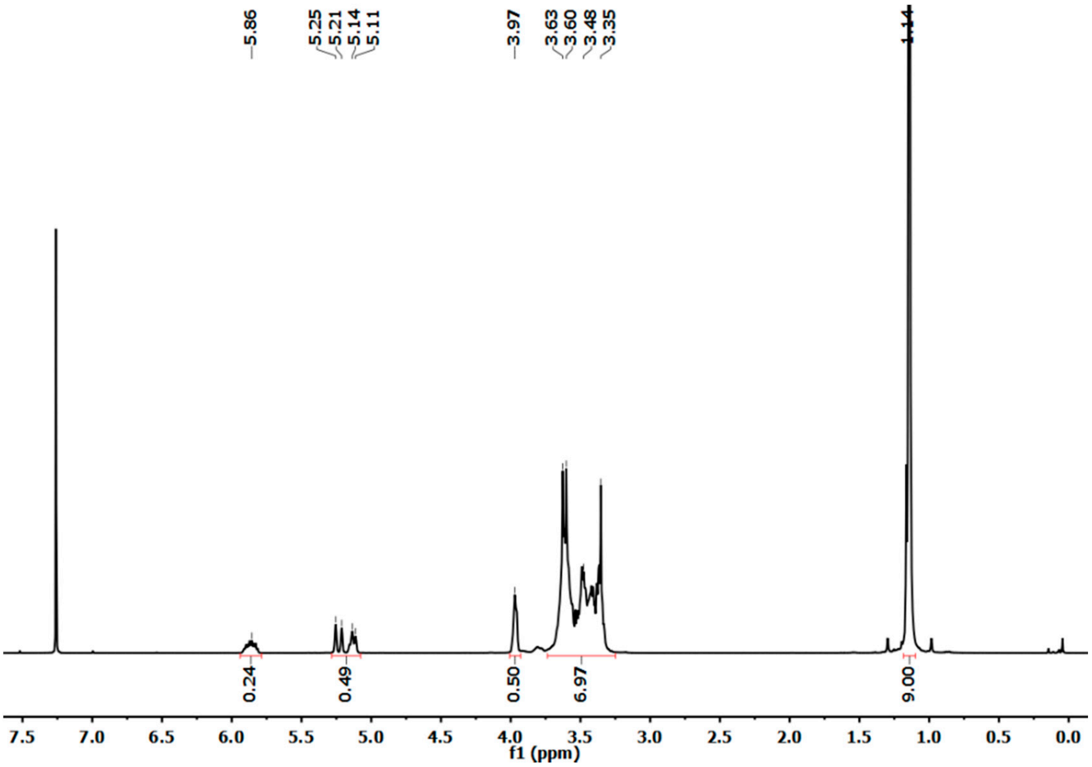

Figure S8. ^{1}H-NMR spectrum (in $CDCl_3$) of P(*t*BGE)$_{0.8}$-*b*-P(AGE)$_{0.2}$ **2**. ^{1}H-NMR (400 MHz, $CDCl_3$): δ = 5.87 (m, 1H, –OCH_2C*H*CH_2), 5.27–5.13 (dd, 2H, –OCH_2CHC*H_2*), 3.98 (d, 2H, –OC*H_2*$CHCH_2$), 3.64–3.38 (m, 10 H, –C*H_2*CHO-$_{(backbone)}$, –OCH_2C*H*O-$_{(backbone)}$, –O–$CH_2CH_{(backbone)}$C*H_2*O–), 3.37 (s, 3H, –O–C*H_3*$_{(Initiator)}$), 1.16 (s, 9H, –O–C(C*H_3*$)_3$) ppm.

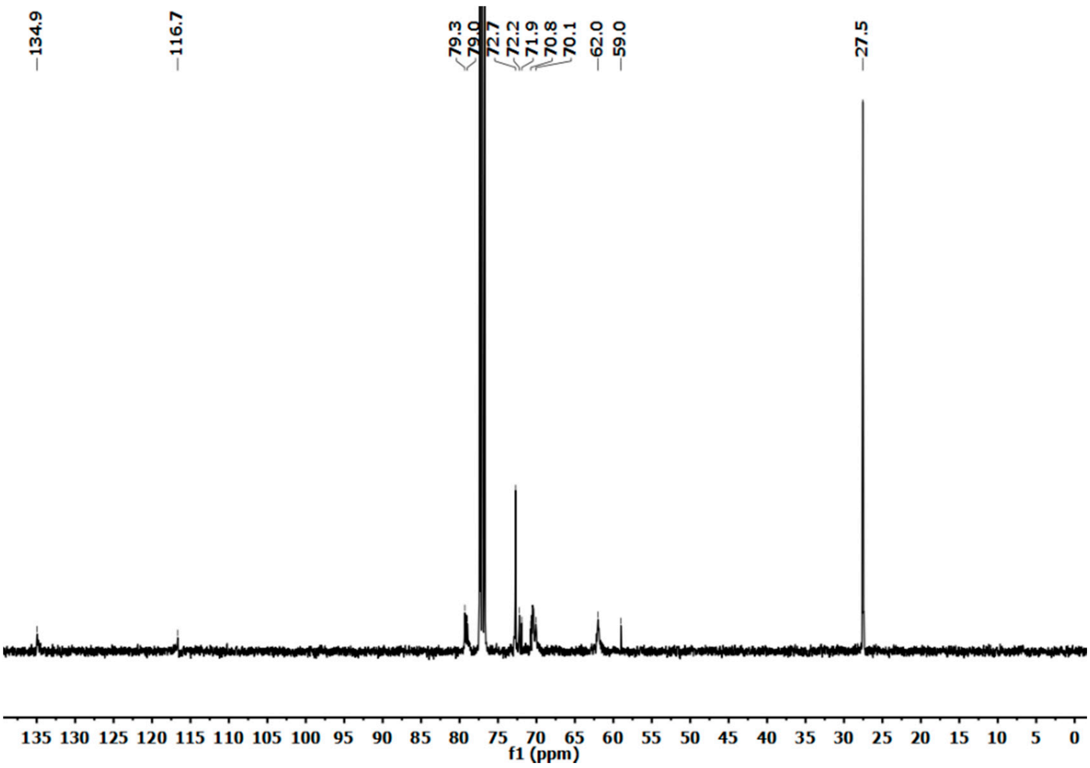

Figure S9. ^{13}C-NMR spectrum (in $CDCl_3$) of P(*t*BGE)$_{0.8}$-*b*-P(AGE)$_{0.2}$ 2. ^{13}C-NMR (100 MHz, $CDCl_3$): δ = 134.9, 116.7, 79.3–79.0, 72.7, 72.2, 71.9, 70.8–70.1, 62.0, 59.0, 27.5 ppm.

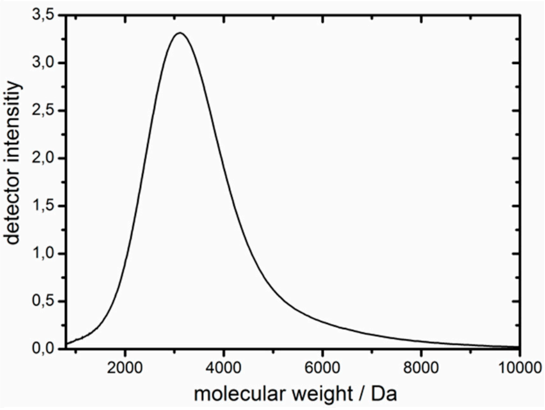

Figure S10. SEC trace (in THF) of P(*t*BGE)$_{0.8}$-*b*-P(AGE)$_{0.2}$ **2**.

6. Synthesis of Polyglycidol Based Microgels

Table S9. Weighed portions for the synthesis of P(EEGE)-*b*-P(AGE) **1** based microgels MG **1** and MG **1.1**.

**Substrate/Reagents**	**for MG 1** [1]	**for MG 1.1** [1]
P(EEGE)-*b*-P(AGE) **1**	1.0	0.25
H_2O	16.0	4.0
toluene	3.48	0.88
dodecylsulfate	0.005	0.002
hexadecane	0.082	0.02
2,2′-(ethylenedioxy)diethanethiol	0.143	0.034
2,2-dimethoxy-2-phenylacetophenone	0.036	0.009
CaLB	0.010	-

[1] numbers represent gram of the corresponding reagent.

Table S10. Weighed portions for the synthesis of P(*t*BGE)-*b*-P(AGE) **2** based microgels MG **2** and MG **2.1**.

Substrate/Reagents	for MG 2 [1]	for MG 2.1 [1]
P(*t*BGE)-*b*-P(AGE) **2**	1.0	0.25
H_2O	16.0	4.0
toluene	3.48	0.88
dodecylsulfate	0.005	0.002
hexadecane	0.082	0.02
2,2′-(ethylenedioxy)diethanethiol	0.16	0.038
2,2-dimethoxy-2-phenylacetophenone	0.037	0.009
CaLB	0.010	-

[1] numbers represent gram of the corresponding reagent.

7. Enzymatic ROP of ε-CL with CaLB Immobilized in Microgels

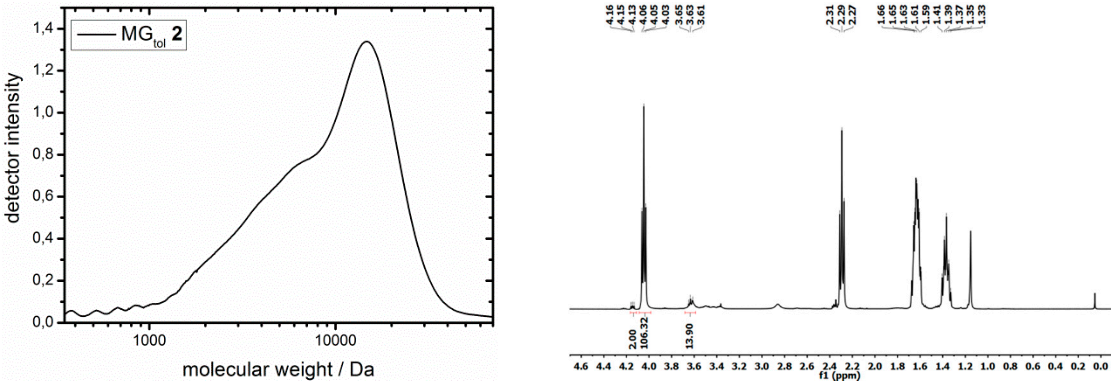

(**a**) (**b**)

Figure S11. (**a**) SEC trace in THF and (**b**) ^{1}H-NMR spectrum in $CDCl_3$ for the enzymatic polymerization of ε-CL with MG_{tol} 2.

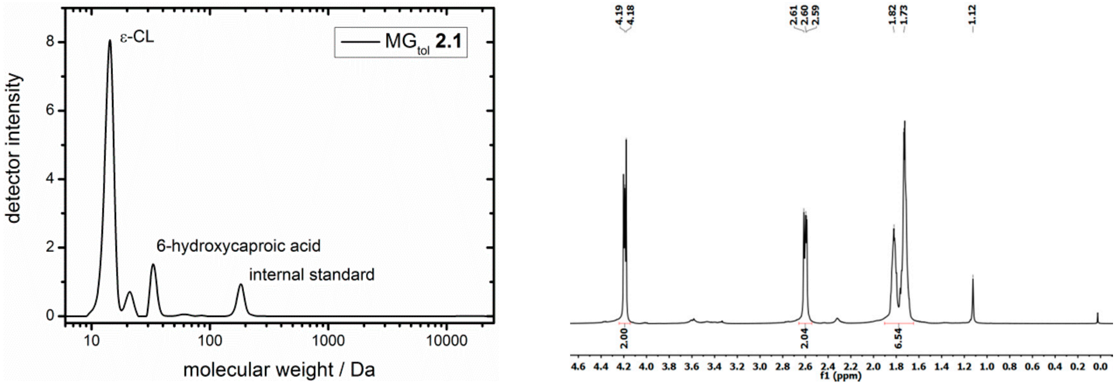

(**a**) (**b**)

Figure S12. (**a**) SEC trace in THF and (**b**) ^{1}H-NMR spectrum in $CDCl_3$ for the enzymatic polymerization of ε-CL with MG_{tol} 2.1.

8. Cloning, Production and Purification of CaLB

All chemicals were of analytical grade or higher quality and purchased from Sigma-Aldrich (Hamburg, Germany), Applichem (Darmstadt, Germany), Carl Roth (Karlsruhe, Germany), or Invitrogen (Darmstadt, Germany). All enzymes were purchased from New England Biolabs GmbH (Frankfurt, Germany), Fermentas GmbH (St. Leon-Rot, Germany) or Sigma-Aldrich (Hamburg, Germany). Thermal cycler (Mastercycler proS; Eppendorf, Hamburg, Germany) and thin-wall PCR tubes (Multi-ultra tubes; 0.2 mL; Carl Roth GmbH, Karlsruhe, Germany) were used in all PCRs. The PCR volume was always 50 μL. The amount of DNA in cloning experiments was quantified using a NanoDrop photometer (ND-1000, NanoDrop Technologies, Wilmington, DE, USA).

Oligonucleotides were purchased from Eurofins MWG operon (Ebersberg, Germany). Plasmid extraction and PCR purification kits were purchased from Macherey-Nagel (Düren, Germany). Omega FLUOstar (BMG LABTECH, Ortenberg, Germany) was used for absorbance detection. Microtiter plates (Greiner Bio-One GmbH, Frickenhausen, Germany) were incubated in a Multitron II Infors shaker (Infors AG, Bottmingen, Switzerland). CaLB gene was synthesized by GeneArt (Regensburg, Germany).

8.1. Cloning of Candida Antarctica Lipase B into the pGAPz Expression Vector

The gene of CaLB was ordered as a synthetic gene and transformed into E. coli DH5α [30]. Plasmid extraction of synthetic gene and pGAPzαA was done with the plasmid DNA purification kit. The CaLB gene was amplified from the vector via PCR with forward Primer (GCTGAAGCTGAATT CTTGCCATCTGGTTCTG) and reverse Primer (CACACTGGGTACCCGTTACTAGTGGATCCG). The PCR product and pGAPzαA were digested using EcoRI (100U) and KpnI (100 U) restriction enzymes. After 20-min heat inactivation at 80 °C and purification of the specific DNA fragments with the PCR purification gel extraction kit, the digested CaLB gene and vector pGAPzαA were ligated using T4 DNA ligase (5 U) resulting in pGAP_CaLB. Plasmid construct was subsequently transformed into E. coli DH5α (purchased from Agilent Technologies; Santa Clara, CA, USA). The plasmid pGAP_CaLB was extracted from E. coli DH5α by using a plasmid extraction kit. About 200 ng plasmid DNA linearized by AVRII was mixed with 80 μL of competent cells, and then it was transformed into Pichia cells (purchased Invitrogen GmbH, Karlsruhe, Germany) by electroporation conducted on Eppendorf Eporator (Eppendorf, Hamburg, Germany) according to the manufacturers instruction P. pastoris transformants via homologous recombination at the GAP promoter region between the transforming DNA and regions of homology within the Pichia genome. Positive clones were initially selected on YPDS plates containing 100 μg/mL Zeocin™ plates.

8.2. Expression of CaLB in 96-well Microtiter Plates

To determine strain with highest lipase production 10 colonies were transferred into 96-well flat-bottom microtiter plates containing 150 μL YPD medium supplemented with Zeocin™ (0.07 μM or 0.1 mg/mL) (master plate). After overnight cultivation in a microtiter plate shaker (30 °C, 900 rpm, 70% humidity), 150 μL main culture (YPD medium without antibiotics) were inoculated with 10 μL preculture (v-bottom MTP, expression plate). The master plate was stored at −80 °C after addition of 100 μL glycerol (30% (*v*/*v*)). Expression plates were cultivated for 24 h (20 °C, 900 rpm, 70% humidity). After expression, v-bottom MTPs were centrifuged for cell harvesting (10 min, 4 °C, 4000*g*). The supernatant was transferred into a new flat-bottom microtiter plate and used for the *p*-nitrophenyl butyrate (*p*NPB) assay.

8.3. p-Nitrophenyl Butyrate (pNPB) Assay in MTP Format for CaLB Activity Measurement

Upon hydrolysis, *para*-nitrophenolate is released and its absorption is detected at 410 nm. The activity of the assay was determined by addition of TEA buffer (90 μL, 100 mm, pH 7.5) to supernatant (10 μL) and freshly prepared substrate solution (TEA buffer (100 μL) containing *p*NPB (0.5 mM) and acetonitrile (10%, *v*/*v*)) in each well. The release of *para*-nitrophenolate was recorded by measuring the absorption at 410 nm at room temperature over 8 min on the microtiter plate reader.

8.4. Production of CaLB in Shake Flask and Purification

Yeast cells pre-grown on YPD agar plate solid medium were inoculated in 10 mL YPD medium and incubated at 30 °C at 200 rpm for 16 h as a pre-culture. The main-culture was inoculated at OD600 of 0.2 and incubated at 20 °C at 220 rpm for 72 h. The supernatant containing the secreted enzyme was separated from the cells by centrifugation (Sorval RC 6; Thermo Fisher Scientific, Waltham, MA, USA) for 30 min at 4 °C, at 4000 rpm.

Tris-acetate buffer (pH 7.2; 250 mM) was added to recovered supernatant in relation 1:10 and filtered with a glass fibre filter (pore size 0.45 μm; GE Healthcare). For enzyme purification first

anion-exchange chromatography was employed, using the ÄKTApilot system (GE Healthcare, München, Germany). The column was packed with 100 mL resin (Fractogel TSK DEAE-650s Merck), a flow rate of 10 mL/min was selected; equilibration with 200 mL tris-acetate buffer (pH 7.2; 25 mM); sample load: 2 L supernatant. The flow through containing the enzyme was collected. In a second purification step hydrophobic interaction chromatography was employed. The column was packed with 100 mL resin (Fractogel TSK Butyl 650 Size S), a flow rate of 10 mL/min was selected; equilibration with 200 mL tris-acetate buffer (pH 7.2; 25 mM) adjusted to 20 mS/cm with ammonium acetate; sample load: 2 L supernatant adjusted to 20 mS/cm with ammonium acetate; wash: 200 mL tris-acetate buffer (pH 7.2; 25 mM, 20 mS/cm) and 100 mL ammonium acetate (0.3 M); elution with a gradient from 0.3 M ammonium acetate to ddH_2O. The sample was lyophilized yielding 100 mg/2000 mL CaLB.

9. Report of Deconvolution of the Molecular Weight Distribution Obtained with Novozym® 435

FIT
Data Points Number: 804
Fitted Curves Number: 5
Parameters Number: 15
Degrees of Freedom (DoF): 789
Data Total Sum of Squares (TSS): 156.8073
Weighting: No

DATA INTERVALS

From	To
0.3491	97.3635

RESULTS
Iterations: 2
Convergence: 7.5447×10^{-11}
Residual Sum of Squares (RSS, χ^2): 0.0566
Reduced RSS: 7.1754×10^{-5}
Residual Standard Deviation: 0.0085
Coefficient of Determination R^2: 0.9996
Adjusted R^2: 0.9996
